# A Cytoplasmic Tail Determinant in HIV-1 Vpu Mediates Targeting of Tetherin for Endosomal Degradation and Counteracts Interferon-Induced Restriction

**DOI:** 10.1371/journal.ppat.1002609

**Published:** 2012-03-29

**Authors:** Tonya Kueck, Stuart J. D. Neil

**Affiliations:** Department of Infectious Disease, King's College London School of Medicine, Guy's Hospital, London, United Kingdom; Fred Hutchinson Cancer Research Center, United States of America

## Abstract

The HIV-1 accessory protein Vpu counteracts tetherin (BST-2/CD317) by preventing its incorporation into virions, reducing its surface expression, and ultimately promoting its degradation. Here we characterize a putative trafficking motif, EXXXLV, in the second alpha helix of the subtype-B Vpu cytoplasmic tail as being required for efficient tetherin antagonism. Mutation of this motif prevents ESCRT-dependent degradation of tetherin/Vpu complexes, tetherin cell surface downregulation, but not its physical interaction with Vpu. Importantly, this motif is required for efficient cell-free virion release from CD4+ T cells, particularly after their exposure to type-1 interferon, indicating that the ability to reduce surface tetherin levels and promote its degradation is important to counteract restriction under conditions that the virus likely encounters *in vivo*. Vpu EXXXLV mutants accumulate with tetherin at the cell surface and in endosomal compartments, but retain the ability to bind both β-TrCP2 and HRS, indicating that this motif is required for a post-binding trafficking event that commits tetherin for ESCRT-dependent degradation and prevents its transit to the plasma membrane and viral budding zones. We further found that while Vpu function is dependent on clathrin, and the entire second alpha helix of the Vpu tail can be functionally complemented by a clathrin adaptor binding peptide derived from HIV-1 Nef, none of the canonical clathrin adaptors nor retromer are required for this process. Finally we show that residual activity of Vpu EXXXLV mutants requires an intact endocytic motif in tetherin, suggesting that physical association of Vpu with tetherin during its recycling may be sufficient to compromise tetherin activity to some degree.

## Author Summary

Tetherin inhibits the release of several diverse enveloped viruses from infected cells and is counteracted by the HIV-1 accessory gene Vpu. Vpu prevents tetherin's incorporation into nascent viral particles, promotes its downregulation from the cell surface and targets tetherin for degradation. Here we identify a determinant that resembles an acidic-dileucine-based sorting sequence in the Vpu cytoplasmic tail that is required for efficient counteraction of tetherin activity, particularly in CD4+ T cells treated with type-1 interferon. Mutation of this motif prevents cell-surface downregulation and degradation of Vpu/tetherin complexes but does not affect their interaction. Rather, in its absence, Vpu accumulates in early endosomes and at the cell surface where it becomes incorporated into assembling virions with tetherin, indicating that this motif modulates sub-cellular trafficking of tetherin. Furthermore Vpu activity is clathrin-dependent and can be reconstituted by replacing a portion of the cytoplasmic tail encompassing this motif with one derived from HIV-1 Nef that is known to bind several clathrin adaptors. Finally, we demonstrate that residual function of the mutant Vpu requires a trafficking motif in tetherin, suggesting that physical interaction of tetherin with Vpu during its recycling to the cell-surface can interfere with its function to a variable extent.

## Introduction

The downregulation of cell-surface immunomodulatory proteins is a common theme in the evasion of innate and adaptive immune responses by mammalian viruses. One host molecule targeted by diverse enveloped viruses is tetherin (CD317/BST-2), an interferon-induced dimeric type-II membrane protein, which inhibits the release of nascent virions from the cell surface [1], [2]. By virtue of an unusual topology that consists of an N-terminal transmembrane domain and a C-terminal glycophosphatidylinositol (GPI)-linkage separated by an extended parallel coiled-coil domain [3]–[6], tetherin partitions into budding virus particles and is thought to directly cross-link the nascent virion to the plasma membrane (PM) [7]. Tethered virions may then be endocytosed and degraded in endosomes. Tetherin itself constitutively recycles between the PM, endosomal and trans-Golgi network (TGN) compartments through a non-canonical trafficking motif (YXYXXV) in its N-terminal cytoplasmic tail, which engages clathrin adaptors AP-1 and AP-2 [8], [9]. Since tetherin targets a structural component of the virion not encoded by the viral genome, namely the host-cell derived membrane, its potential importance in the innate antiviral response is highlighted by multiple examples of virally-encoded countermeasures (reviewed in [10]).

The ability to counteract tetherin is conserved among human and simian immunodeficiency viruses (HIVs/SIVs), although the viral proteins tasked with this activity vary [10]. In HIV-1 the accessory protein Vpu fulfills this role. Vpu, a small integral membrane phosphoprotein, directly associates with tetherin through interactions between the transmembrane domains of both proteins [11]–[14]. Vpu blocks tetherin incorporation into assembling virions [7] and leads to a reduction of tetherin levels on the plasma membrane (PM) [2]. Subsequently, tetherin is degraded, most likely in lysosomal compartments, by an ubiquitin-dependent mechanism [15], [16]. Phosphorylation of Vpu on two conserved serine residues recruits a SCF β-TrCP1/2 E3 ligase complex [17] that ubiquitinates the tetherin cytoplasmic tail on multiple residues [18], and recent evidence demonstrates that this tetherin degradation is dependent on the ESCRT pathway [19]. However, tetherin degradation is not strictly required for Vpu activity [20], [21]. While recruitment of the ESCRT-0 subunit HRS by Vpu counteracts tetherin activity [19], a novel core component of ESCRT-1, Ubiquitin Associated Protein 1 (UBAP1), essential for tetherin degradation induced by both Vpu and the KSHV ubiquitin ligase K5, is dispensable [22]. Coupled with recent evidence that dysregulation of the entire late endosomal compartment by mutants of Rab7a [23], this suggests an emerging picture that Vpu alters tetherin trafficking to counteract its antiviral activity prior to lysosomal delivery. While HIV-2 and SIV tetherin antagonists Env and Nef promote tetherin internalization through their interactions with AP-2, [24]–[26], Vpu does not enhance the rate of tetherin endocytosis [13], [16]. Rather it is thought that Vpu/tetherin interactions preclude both the recycling of tetherin back to the cell surface and the transit of newly synthesized tetherin to the PM by trapping it in intracellular compartments, notably the TGN [13]. Consistent with this, the ability of Vpu to localize to the TGN correlates with tetherin antagonism [27], and disruption of the recycling compartment by a dominant Rab11a mutant compromises Vpu activity [28]. Truncations of the Vpu cytoplasmic tail, particularly the second alpha helix, lead to aberrant localization and a reduction in its anti-tetherin activity, suggesting it harbors a domain required for Vpu function [29].

In this study we have examined the role of the second alpha helix of HIV-1 Vpu in tetherin antagonism. We identify a putative sorting signal that is required for post-binding trafficking of Vpu/tetherin complexes and inhibition of antiviral activity in primary CD4+ T cells. While this signal can be functionally replaced by a clathrin adaptor binding peptide derived form HIV-1 Nef, Vpu activity does not require the canonical adaptors AP-1, AP-2 or AP-3. Moreover, because residual activity of second helix mutants requires an intact recycling signal in tetherin, we propose that second alpha helix mutants are selectively defective for routing tetherin into an endosomal degradation pathway thereby inhibiting its transit to the PM and incorporation into nascent virions.

## Results

### Determinants of tetherin inactivation in the second alpha helix of the Vpu cytoplasmic tail

**Figure 1:**
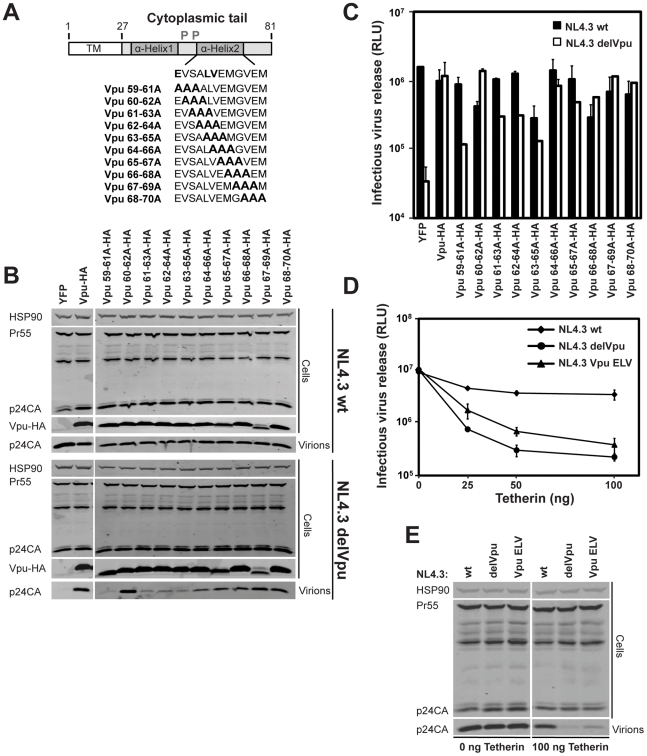
E59, L63 and V64 in the second alpha helix of the Vpu cytoplasmic tail are required to efficiently counteract tetherin. (**A**) Schematic representation of alanine scan mutagenesis in the second alpha helix of the codon-optimized HIV-1 NL4.3 Vpu protein. (**B**) 293T cells were transfected with NL4.3 wt or NL4.3 delVpu proviruses in combination with tetherin and the indicated pCR3.1 Vpu-HA expression vectors. 48 h post transfection, cell lysates and pelleted supernatant virions were harvested and subjected to SDS-PAGE and analyzed by Western blotting for HIV-1 p24CA, Vpu-HA and Hsp90 serving as loading control, and analyzed by LiCor quantitative imager. (**C**) Viral supernatants from **B** were assayed for infectivity using HeLa-TZMbl reporter cells. Infectious virus release is plotted on a log scale as β-galactosidase activity in relative light units (RLU). Error bars represent standard deviations of the means of three independent experiments. (**D**) E59A, L63A and V64A mutations were inserted into the *vpu* gene of the NL4.3 provirus referred to as NL4.3 Vpu ELV. 293T cells were transfected with NL4.3 wt, NL4.3 delVpu or NL4.3 Vpu ELV proviral plasmids together with increasing doses of tetherin expression vector. The resulting infectivity was determined as in **C**, error bars represent standard deviations of the means of three independent experiments. (**E**) Cell lysates and pelleted viral supernatants from 0 ng and 100 ng tetherin input from **D** were subjected to SDS-PAGE and analyzed by Western blotting for HIV-1 p24CA and Hsp90, and analyzed by LiCor quantitative imager.

Truncations of the Vpu cytoplasmic tail lead to aberrant localization and a reduction in its anti-tetherin activity [29]. To further study the determinants within the second alpha helix of Vpu that account for TGN localization, we performed overlapping triple-alanine scan mutagenesis through the second alpha helix of a codon optimized HIV-1 NL4.3 Vpu construct bearing a C-terminal HA tag (Figure 1A). We then assayed these Vpu mutants for their ability to rescue Vpu-defective HIV-1 from tetherin restriction. 293T cells were transfected with wildtype HIV-1 (HIV-1 wt) or Vpu defective HIV-1 (HIV-1 delVpu) proviruses in combination with fixed doses of a human tetherin expression vector, and 25 ng of Vpu-HA or mutant thereof. 48 h after transfection cell lysates and supernatants were harvested and analyzed for physical viral yield by Western blot (Figure 1B) or supernatant infectivity of HeLa-TZMbl indicator cells (Figure 1C). As expected, in the absence of Vpu, both supernatant particle yield and infectivity of HIV-1 delVpu was profoundly reduced in the presence of tetherin, expression of Vpu *in trans* rescued virus production to wildtype levels. By contrast mutations encompassing either E59 or L63 and V64 but not the intervening or subsequent amino acids displayed defective rescue of HIV-1 delVpu (Figure 1B and C). All Vpu mutants with the exception of Vpu 67-69A-HA were expressed equivalently. Vpu 63-65A-HA appeared to display a dominant interfering activity on HIV-1wt titer, but this was not reflected as apparently in particle yield. Thus these data suggested a functional requirement for E59 and L63/V64 in tetherin antagonism by Vpu. To confirm this we mutated these residues to alanine in the context of an HIV-1 NL4.3 provirus (NL4.3 Vpu ELV) and examined viral release from 293T cells in the presence of increasing expression of tetherin. Because this part of Vpu overlaps with start of the Env open reading frame in the provirus, these mutations were rendered silent in the +1 reading frame and displayed no defect in virus release in the absence of tetherin (Figure 1D and E). In agreement with the virus rescue *in trans*, NL4.3 Vpu ELV release was markedly defective in the presence of increasing tetherin doses, although it did display a residual antagonism of tetherin when compared to the full Vpu-defective deletion (Figure 1D and E).

**Figure 2:**
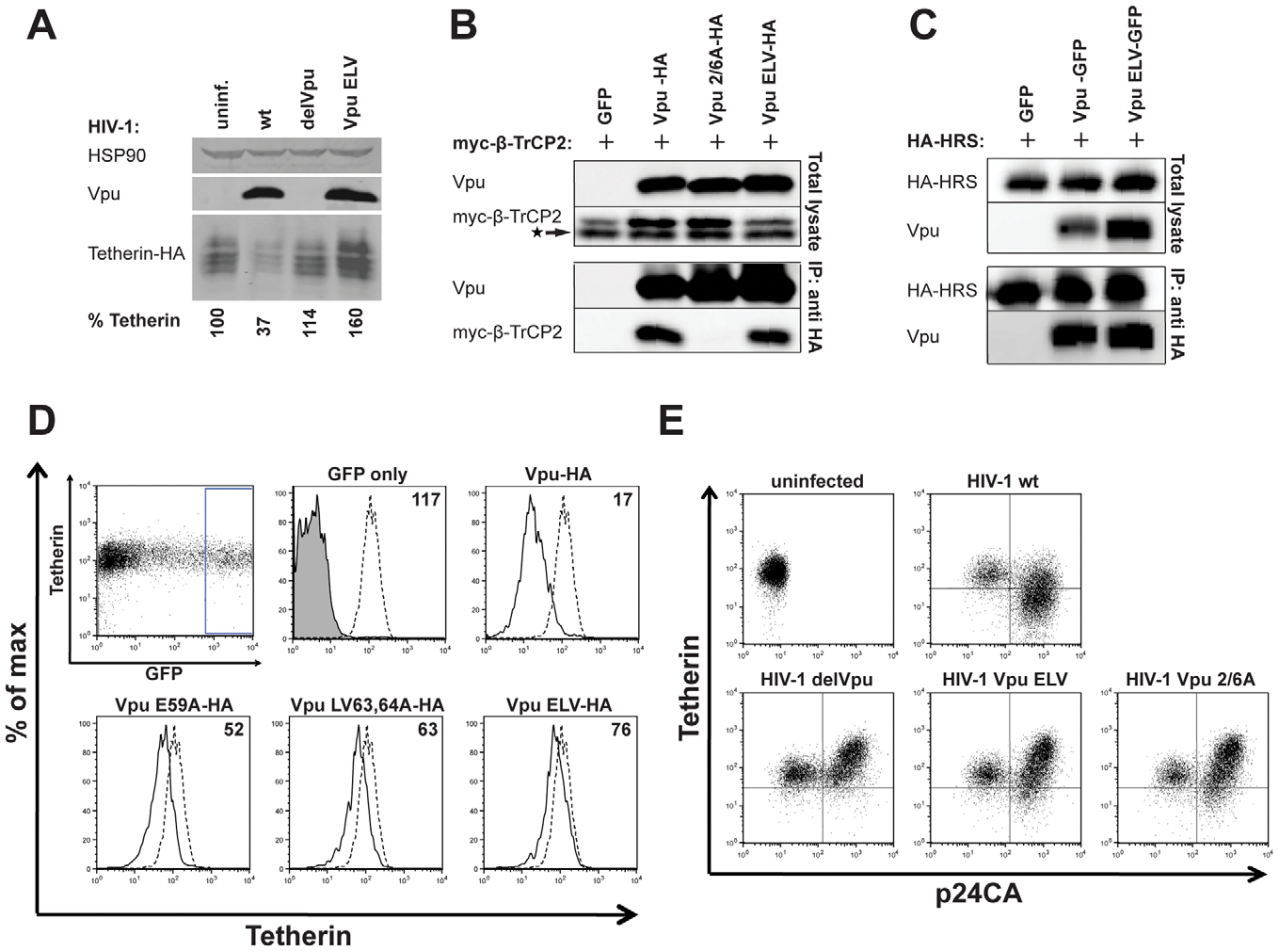
Vpu ELV mutants are defective for tetherin degradation and cell-surface downregulation. (**A**) HT1080 cells stably expressing tetherin-HA were infected with VSV-G-pseudotyped HIV-1 wt, HIV-1 delVpu or HIV-1 Vpu ELV at an MOI of 2. 48 h post infection, cells were harvested and subjected to SDS-PAGE and analyzed by Western blotting for tetherin-HA, Vpu and Hsp90, and analyzed by LiCor quantitative imager. Relative tetherin-HA levels are indicated below each lane. The blot shown is a representative example of 3 independent experiments. (**B**) 293T cells were transfected with pCR3.1 Vpu-HA, Vpu 2/6A-HA, or Vpu ELV-HA in combination with pCR3.1 myc-β-TrCP2. 48 h post transfection, cells were lysed and immunoprecipitated with anti-HA antibody. Lysates and precipitates were subjected to SDS-PAGE and analyzed by Western blotting for Vpu and myc-β-TrCP2, and analyzed by ImageQuant. The star represents an unspecific band. (**C**) Similarly, 293T cells were transfected with pCR3.1 HA-HRS in combination with Vpu-GFP or Vpu ELV-GFP expression constructs. Cell lysates were precipitated with an anti-HA antibody and analyzed as in **C**. (**D**) HeLa cells were co-transfected with pCR3.1 Vpu-HA or indicated Vpu mutant in combination with a GFP expression construct. Cell surface staining for endogenous tetherin was analyzed by flow cytometry 48 h post transfection. GFP positive cells were gated and tetherin levels (solid lines) were compared to those of untransfected HeLa cells (dotted lines). Numbers indicate median fluorescence intensities of surface tetherin on transfected cells. The solid peak in the upper middle histogram represents the binding of the isotype control. (**E**) Jurkat cells were infected with VSV-G-pseudotyped HIV-1 wt, HIV-1 delVpu, HIV-1 Vpu ELV or HIV-1 Vpu 2/6A at an MOI of 1. 48 h post infection, cells were stained for cell surface tetherin and intracellular p24CA, and analyzed by flow cytometry. Productively infected cells were identified by comparing with culture infected with the same MOI in the presence of 50 µM AZT to control for p24CA uptake of the inoculum (Figure S1C).

We next examined the phenotypic basis for the defect in tetherin antagonism by Vpu ELV. Vpu stimulates the ubiquitin-dependent degradation of tetherin, most likely in lysozomal compartments. We infected HT1080 cells stably expressing human tetherin bearing an HA-tag in the extracellular domain (HT1080/tetherin-HA) with VSV-G-pseudotyped HIV-1 wt, HIV-1 delVpu or HIV-1 Vpu ELV at an MOI of 2 to ensure >90% cell infection. 48 h later the cells were lysed and Western blotting performed for relative tetherin-HA levels (Figure 2A). As expected, cells infected with HIV-1 wt showed reduced steady state levels of tetherin that was not apparent in those infected with HIV-1 delVpu. Similarly, in cells infected with HIV-1 Vpu ELV there was no evidence of tetherin degradation, but interestingly there appeared to be enhanced levels of tetherin, perhaps suggesting stabilization of the protein in the presence of the mutant Vpu. Thus E59, L63, V64 mutations abolish the ability of Vpu to induce tetherin degradation. Since this degradation is dependent on Vpu binding to β-TrCP2 via a phosphorylated pair of serines (S52 and S56) [16], [17], [30], we tested whether Vpu ELV mutants were defective for interaction with β-TrCP2 in co-immunoprecipitations from transfected cells (Figure 2B). β-TrCP2 was co-immunoprecipitated with Vpu-HA and Vpu ELV-HA, but as expected, not the phospho-mutant Vpu 2/6-HA, ruling out this defect in Vpu ELV.

Recent data suggests that ESCRT-mediated degradation of tetherin in the presence of Vpu is mediated by interaction of Vpu with HRS (ESCRT-0) [19]. We could further show that both Vpu and Vpu ELV also co-precipitated with HA-HRS from transfected 293T cells (Figure 2C) indicating that an inability to recruit ESCRT-0 does not explain the defect in Vpu ELV-mediated degradation of tetherin.

We then examined the ability of Vpu ELV mutants to downregulate surface tetherin levels. We first transfected HeLa cells (that express tetherin constitutively) with Vpu-HA expression vectors in combination with a GFP reporter. 48 h later surface tetherin was assayed by flow cytometry in the GFP positive cells (Figure 2D). As expected, wildtype Vpu expression reduced cell surface tetherin levels. Vpu mutants bearing E59A, LV63,64A mutations, or the full ELV mutant all displayed a reduced capacity to downregulate surface tetherin levels (Figure 2D and S1A). To confirm this in a relevant cell-type, we then infected CD4 positive Jurkat T cells with HIV-1 wt, HIV-1 delVpu, HIV-1 Vpu ELV or HIV-1 Vpu 2/6A. 48 h later, the cells were stained for surface tetherin and co-stained for intracellular p24CA as a marker of infection (Figure 2E). To discriminate between truly infected cells, and those that acquired p24+ debris by exposure of cells to high titre (MOI 1) of viral inoculum, we compared to cells exposed to virus in the presence of 50 µM AZT (Figure S1C). Cultures infected with HIV-1 wt showed clear downregulation of tetherin on the surface of p24CA positive cells. By contrast, tetherin was not downregulated from the surface of either HIV-1 delVpu, HIV-1 Vpu 2/6A or HIV-1 Vpu ELV infected cells. Rather, tetherin levels were raised on some infected cells, perhaps reflecting accumulation of tethered virions on the cell surface. A similar result was observed for HeLa cells infected with the same viral stocks (Figure S1B), although in this case we could detect no enhanced tetherin surface expression on cells infected with Vpu mutants, likely due to their endocytic removal from the cell surface [31].

### Vpu ELV mutants localize to early endosomal compartments and the cell surface

**Figure 3:**
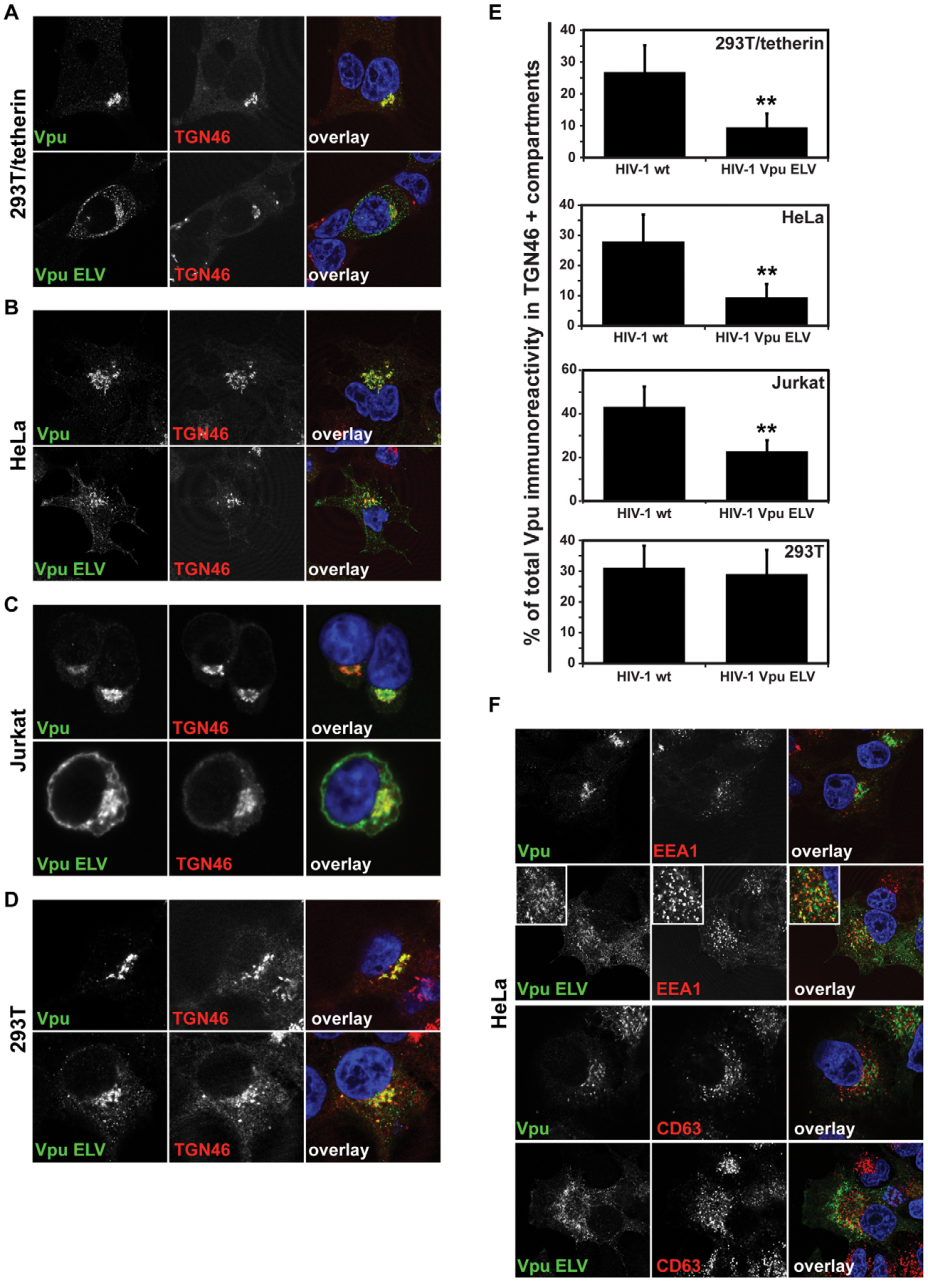
Vpu ELV mutants localize to early endosomal compartments in tetherin-expressing cells. 293T/tetherin (**A**), HeLa (**B**), Jurkat (**C**) or parental 293T (**D**) were infected with HIV-1 wt or HIV-1 Vpu ELV at an MOI of 1. 48 h later the cells were fixed and stained for Vpu (green) and the TGN marker TGN46 (red) and examined by confocal microscopy. Panels are of representative examples. (**E**) The percentage of the total Vpu immunoreactivity localized to TGN46+ compartments was calculated for cells (n = 20) from **A–D** using the Leica Confocal Software. Results were analyzed by unpaired 2-tailed t-test - ** P = 10^−8^ or lower. (**F**) HeLa cells as in **B** were stained for Vpu (green) and the early endosomal marker EEA1 or late endosomal marker CD63 (red).

The E_59_XXXL_63_V_64_ motif in Vpu resembles an acidic dileucine sorting signal (D/E)XXXL(L/I/M/V) found in the cytoplasmic tails of membrane proteins that traffic through endosomal compartments (reviewed in [32]). We therefore addressed whether mutation of this motif affected Vpu subcellular localization. To this end we infected 293T expressing tetherin or not, as well as Jurkat and HeLa cells with VSV-G-pseudotyped HIV-1 NL4.3 and NL4.3 Vpu ELV and stained them for Vpu in combination with several subcellular markers 48 h later. As expected, in all cells, the predominant localization of wildtype Vpu was in association with the TGN, with between 20–50% of the Vpu immunoreactivity visible in TGN46+ compartments (Figure 3A–D). The proportion of the Vpu ELV mutant in TGN46+ compartments was significantly reduced in 293T/tetherin, Jurkat and HeLa, and appeared as “endosome-like” puncta in the cytoplasm and associated on or near the plasma membrane (Figure 3A–C and E). Interestingly in the parental 293T cells, which lack tetherin expression, Vpu and Vpu ELV localization was indistinguishable, and predominantly associated with TGN46+ compartments (Figure 3D and E). Thus the difference in Vpu ELV localization appeared to be tetherin-dependent. The nature of these extra-TGN compartments was further analyzed in HeLa cells and revealed that Vpu ELV accumulated in EEA1+ early/sorting endosomal compartments, but not CD63+ late endosomes (Figure 3F). Thus mutation of the EXXXLV motif leads to endosomal and surface localization of Vpu consistent with it being required for modulating the trafficking of tetherin.

### Vpu ELV mutants interact with tetherin in infected cells and are incorporated into nascent virions in a tetherin-dependent manner

**Figure 4:**
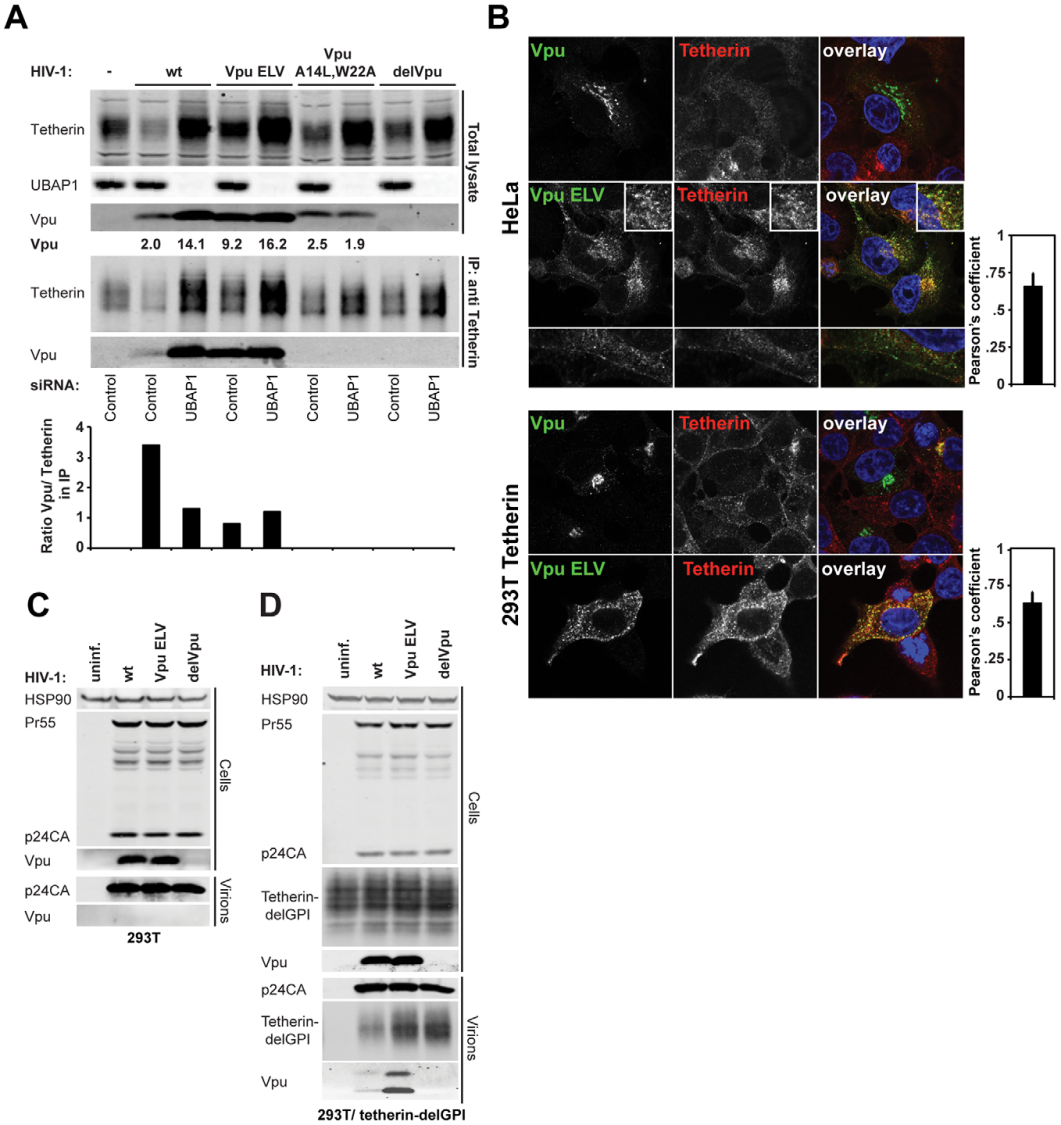
Vpu ELV interacts and colocalizes with tetherin, and is incorporated into nascent virions. (**A**) 293T/tetherin cells were transfected twice over 48 h with siRNA oligonucleotide directed against UBAP1 or Non-targeting control. The cells were then infected with the indicated virus at an MOI of 2. 48 h later cell lysates were immunoprecipitated with an anti-tetherin monoclonal antibody. Lysates and immunoprecipitates were separated by SDS-PAGE and blotted for tetherin, UBAP1 or Vpu using LiCor quantitative Western blotting. Numbers under the Vpu lanes of the cell lysate represent relative band intensities. The histogram below the IP represents the ratio of tetherin band intensity to that of Vpu in the co-IP. (**B**) HeLa and 293T/tetherin cells were infected as in Figure 3 and stained for Vpu (green) and tetherin (red) and examined by confocal microscopy. Adjacent histograms quantify the degree of co-localization of Vpu ELV and tetherin (Pearson's Correlation Coefficient calculated using ImageJ) for 20 individual cells. (**C**) and (**D**) 293T or 293T cells stably expressing tetherin delGPI were infected with HIV-1 wt, HIV-1 Vpu ELV or HIV-1 delVpu at an MOI of 1. 48 h post infection, cells were harvested and viral supernatants were pelleted through a 20% sucrose cushion. Cells and virions were subjected to SDS-PAGE and Western blotting for tetherin delGPI, Vpu, HIV-1 p24CA and Hsp90 and analyzed by LiCor quantitative imager.

As a putative trafficking signal, the EXXXLV motif may exert its effect on tetherin antagonism in two ways. Firstly, the sequence may be required to permit Vpu to traffic to a compartment where it can interact with tetherin; secondly, the sequence may be required for post-interaction trafficking of Vpu/tetherin complexes such that tetherin is not incorporated into budding virions and expression on the cell surface is reduced. A potential confounding factor in investigating however is that wildtype Vpu induces tetherin degradation, while Vpu ELV does not. Whilst a Vpu 2/6 control maybe applicable, controversies surrounding the nature of its phenotype in terms of tetherin counteraction also make it problematic. To alleviate these issues, we took advantage of our recent observations with novel ESCRT-I component, UBAP1 [22]. UBAP1 contains 3-tandem ubiquitin-binding domains and is found in complex with the core ESCRT-I components TSG101, VPS28 and VPS36. However unlike them, UBAP1 is only required for ESCRT-dependent endosomal degradation and not viral assembly or cytokinesis [22], [33]. Interestingly, UBAP1 is essential for both Vpu and K5-mediated degradation of tetherin, but is not required for Vpu-mediated tetherin antagonism, implying that commitment of tetherin into a degradative pathway by Vpu, but not ESCRT-I function itself, counteracts tetherin activity [22]. We therefore first examined whether Vpu or Vpu ELV interacted with tetherin in immunoprecipitations in the presence or absence of siRNA-mediated silencing of UBAP1 by quantitative Western blotting (Figure 4A). 293T/tetherin cells were transfected twice over 48 h with either UBAP1 or control siRNA, before being infected with HIV-1 wt, HIV-1 Vpu ELV, HIV-1 delVpu, or HIV-1 Vpu A14L/W22A that contains a Vpu transmembrane mutation that abolishes tetherin interaction [14]. As expected, while tetherin levels were reduced in HIV-1 wt infected cells, they were unaffected by Vpu-defective or A14L/W22A mutants and, as in Figure 2, stabilized in cells infected with the Vpu ELV mutant. Furthermore, as expected, UBAP1 siRNA treatment rescued tetherin levels from Vpu-mediated degradation, but also further enhanced total cellular levels of tetherin [22] consistent with the known role of ESCRT in tetherin's natural turnover [19]. Interestingly, UBAP1 siRNA also enhanced the total cellular content of wildtype Vpu to that of the Vpu ELV mutant (approximately 4–5 fold). Immunoprecipitation of tetherin from the lysates from these cells revealed a similar picture. Wildtype Vpu was detected associated with residual tetherin precipitated from cells, and this was markedly increased upon UBAP1 knockdown. This data strongly suggests that Vpu itself may be co-degraded with tetherin in endosomal compartments. The Vpu ELV mutant efficiently co-precipitated with tetherin irrespective of UBAP1 knockdown, and as expected the A14L/W22A failed to co-precipitate under either condition. Interestingly, however, the ratio of relative band intensities between of Vpu or Vpu ELV precipitated with tetherin in the presence UBAP1 siRNA was equivalent, indicating that Vpu ELV was not defective for physical tetherin interaction, suggesting that the Vpu ELV mutant's defect in tetherin antagonism is due to an inability to mediate post-binding trafficking of Vpu/tetherin complexes into an ESCRT-dependent pathway in which both proteins are degraded. Consistent with this notion, Vpu ELV co-localized with tetherin in infected HeLa and 293T/tetherin, both in peripheral endosomal structures and at the cell surface (Figure 4B). By contrast, the little tetherin visible in cells infected with wildtype virus co-localized with Vpu in perinuclear areas.

Vpu is not a constituent of HIV-1 particles. We therefore reasoned that if the EXXXLV motif was required to prevent tetherin trafficking to viral budding sites and commit it for degradation, interaction with tetherin itself might lead to Vpu ELV mutant accumulation in nascent viral particles. To test this hypothesis we took advantage of a tetherin mutant lacking its GPI anchor (tetherin-delGPI), which despite its high surface expression, does not restrict virus particle release, but accumulates in virus particles and is sensitive to Vpu [7]. 293T/tetherin-delGPI cells were mock-transfected or transfected with NL4.3 wt, NL4.3 Vpu ELV, and NL4.3 delVpu. 48 h later cell supernatants were centrifuged through a sucrose cushion and analyzed for tetherin incorporation (Figure 4C and D). As expected, high levels of tetherin-delGPI could be detected in NL4.3 delVpu viral pellets, but not in pelleted supernatants from mock-transfected cells. Tetherin-delGPI incorporation was reduced in the wildtype virus consistent with tetherin removal from the cell surface. The level of tetherin incorporation in NL4.3 Vpu ELV particles was similar to that of the Vpu-defective mutant. Interestingly, NL4.3 Vpu ELV particles contained detectable levels of Vpu (in this case Vpu appears as a doublet band which we suggest may be due to exposure to active HIV-1 protease in the particle). By contrast, no Vpu was detectable in any viral particles derived from 293T cells, indicating that Vpu ELV incorporation into viral particles was tetherin-dependent. Taken together these data indicate that the EXXXLV motif is required for efficient tetherin antagonism, by modulating the trafficking of tetherin such that it cannot become efficiently incorporated into nascent viral particles.

### The second alpha helix of Vpu can be complemented by a D/EXXXLL-containing peptide derived from HIV-1 Nef

Acidic-dileucine based sorting signals, D/EXXXL(L/I), act as binding sites for a hemicomplex of sigma and adaptin subunits of the canonical clathrin adaptors AP-1, AP-2 and AP-3, and are required for endocytic and endosomal/Golgi trafficking of these proteins [32]. While the requirement for the acidic and first leucine residues are absolute, the third position is less well conserved, and can be L, I or on occasion V or M. Analysis of the cytoplasmic tails of Vpu sequences from most clades of HIV-1 group M, show that a putative EXXXL(V/M/I) is well conserved in the second alpha helix (Figure S2A). In contrast to other HIV-1 subgroups, Clade C and F isolates have an EXXXLL motif juxtaposed to the plasma membrane in helix 1 (not shown), which has been previously suggested to be a determinant of Clade C Vpu localization to the PM [34]. In subgroup B, the V64 position is usually V or M, although occasional I or L residues are found at this position. We mutated position 64 to M, L or I in NL4.3 Vpu and found no defect in these proteins' ability to counteract or downregulate tetherin from the surface (Figure S2B and S2C), in agreement with the above data demonstrating that this position is the least important of the three, and consistent with the role of this motif as a sorting signal.

**Figure 5:**
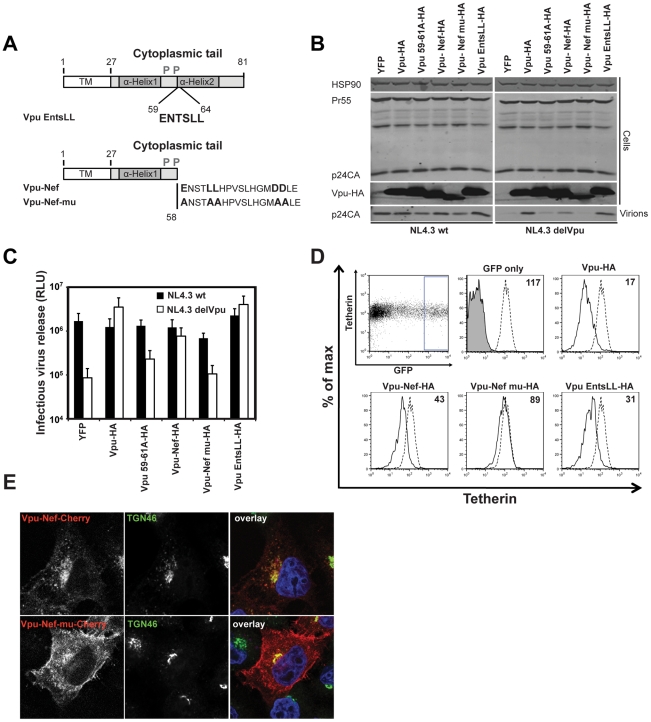
The second alpha helix of Vpu can be functionally replaced by the D/EXXXLL endocytic signal from HIV-1 Nef. (**A**) Schematic representation of Vpu ENTSLL and Vpu-Nef chimeric constructs. (**B**) 293T cells were transfected with NL4.3 wt of NL4.3 delVpu proviral plasmids in combination with tetherin and indicated pCR3.1 Vpu-HA or Vpu-Nef-HA chimera. 48 h post transfection, cell lysates and pelleted supernatant virions were harvested and subjected to SDS-PAGE and analyzed by Western blotting for HIV-1 p24CA, Vpu-HA and Hsp90, and analyzed by LiCor quantitative imager. (**C**) Infectivity of viral supernatants from **B** was determined on HeLa-TZMbl cells as in Figure 1B. Error bars represent standard deviation of three independent experiments. (**D**) HeLa cells were co-transfected with Vpu or indicated Vpu mutant and a GFP expression construct. Cell surface staining for endogenous tetherin was analyzed by flow cytometry 48 h post transfection, as in Figure 2D. (**E**) HeLa cells were transfected with Vpu-Nef or Vpu-Nef-mu CherryFP fusions (red) and counterstained for TGN46 (green) and DAPI (blue) and examined by confocal microscopy.

The Nef proteins of primate immunodeficiency viruses are also multifunctional adaptor proteins, targeting a variety of immunoregulatory cell surface molecules for downregulation and degradation [35]. Nef interacts promiscuously with AP-1, AP-2 and AP-3 through a conserved C-terminal EXXXLL motif [36], [37]. The interaction of AP-2 with this site is essential for Nef targeting of CD4 for ESCRT-dependent lysozomal degradation [37], but Nef-mediated downmodulation of Class I MHC molecules requires AP-1 [38]. Importantly several SIV Nef proteins are also tetherin antagonists [39], [40] and again this is dependent on the EXXXLL motif [26]. We therefore asked whether the C-terminus of HIV-1 Vpu could be functionally substituted with a known AP-binding site from these proteins. The EVSALV motif of NL4.3 Vpu was first replaced with the core AP-binding site from NL4.3 Nef, ENTSLL (Figure 5A). Since this is similar to sites already tested in the previous experiment shown in Figure S2, this Vpu was as functional as the wildtype protein in virus rescue experiments (Figure 5B and C). We then replaced the entire cytoplasmic tail of Vpu from residue 58 with a 19 amino acid stretch derived from Nef including the ENTSLL and a downstream dual-aspartic acid motif that has been previously shown to stabilize AP-2 interactions [41]. Remarkably, the Vpu/Nef chimeric protein substantially recovered tetherin antagonistic activity (Figure 5B and C). Moreover, this chimera also displayed improved tetherin downregulation from the surface of transfected HeLa cells (Figure 5D). This activity was entirely dependent on the key amino acids required for AP-interaction as a chimera in which E, LL and DD positions were mutated to alanine was unable to counteract tetherin or downregulate it from the surface (Figure 5B–D). Examination of the subcellular distribution of Vpu-Nef or the mutant fused to CherryFP suggested that the mutant was localized more prominently to the PM consistent with a defect in trafficking imparted by the mutation (Figure 5E). Thus Vpu function can be substantially recovered by replacing its entire second alpha helix with a promiscuous AP-binding (D/E)XXXL(L/I) sorting signal, indicating that linking Vpu directly to the clathrin trafficking machinery can restore its activity in absence of the second alpha helix.

### Tetherin antagonism by Vpu is clathrin-dependent, but independent of canonical adaptor proteins that bind acidic di-leucine motifs

**Figure 6:**
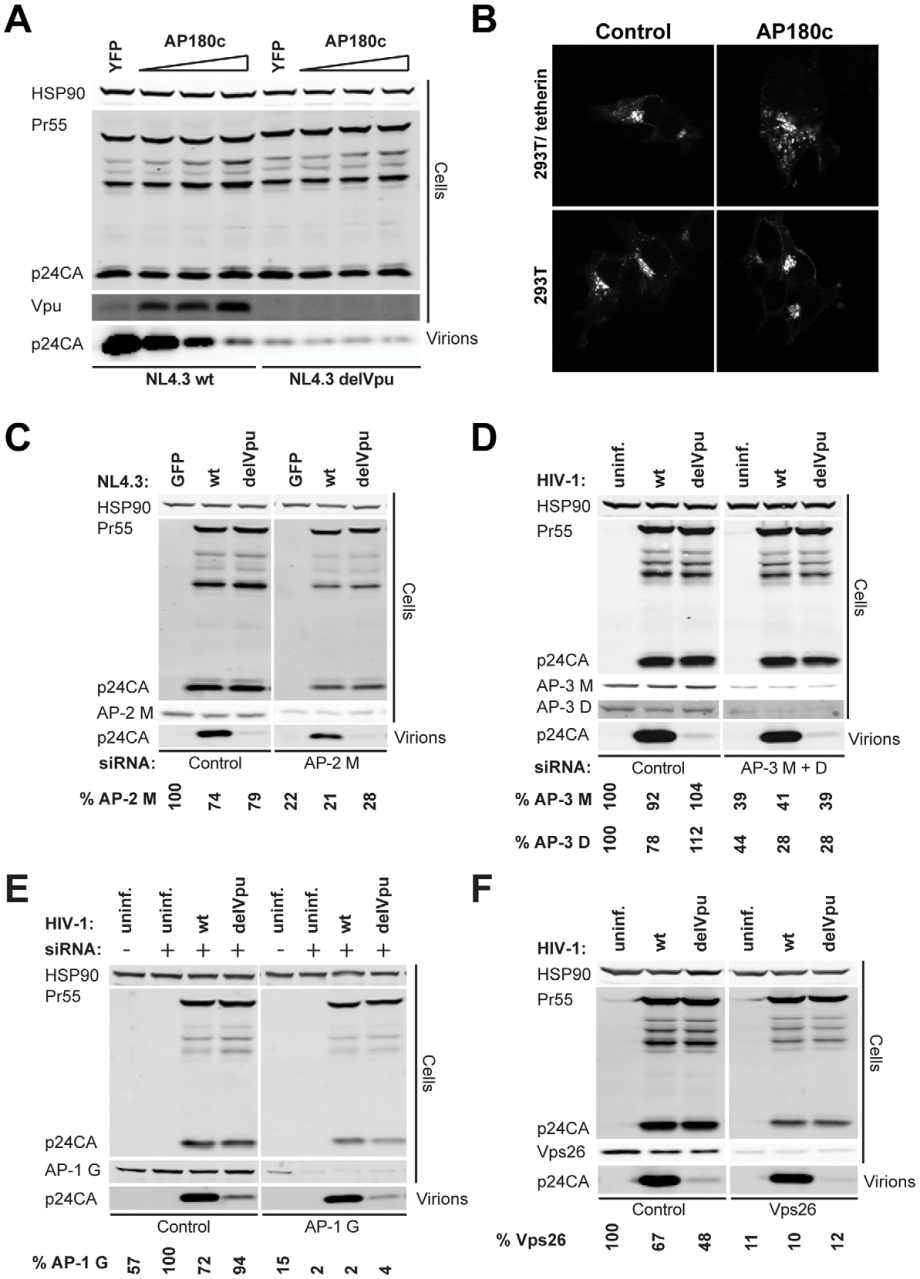
AP180c inhibits Vpu-mediated tetherin antagonism but AP-1, AP-2, AP-3 and retromer are dispensable. (**A**) 293T/tetherin were transfected with NL4.3 wt or NL4.3 delVpu proviral plasmids in combination with either YFP or increasing doses of an AP180c expression vector. 48 h post transfection, cell lysates and pelleted supernatant virions were harvested and subjected to SDS-PAGE and analyzed by Western blotting for HIV-1 p24CA, Vpu and Hsp90 serving as loading control, and analyzed by LiCor quantitative imager. (**B**) 293T or 293T/tetherin transfected with pCR3.1 Vpu-YFP with or without AP180c co-expression were fixed and imaged after 48 h (**C**) 293T cells stably expressing tetherin were transfected twice with pooled control or AP-2μ1 siRNAs and co-transfected with NL4.3 wt, NL4.3 delVpu or GFP expression vectors. Cell lysates and supernatants were analyzed by Western blotting 48 h later as described in Figure 1B. (**D**) 293T cells stably expressing tetherin were transfected twice with pooled control or siRNA pools against AP-3δ1 and AP-3μ1. 4 h post the second transfection the cells were infected with VSV-G-pseudotyped HIV-1 wt or HIV-1 delVpu virus stock at an MOI of 1. Cell lysates and supernatants were analyzed by Western blotting 48 h later as described in Figure 1B. (**E**) HeLa cells expressing a doxycycline-inducible shRNA hairpin against AP-1γ1were transfected twice with pooled control or siRNA pools against AP-1γ1. The cells were infected and analyzed as in **C**. (**F**) 293T cells stably expressing tetherin were transfected twice with pooled control or siRNA pools against the retromer subunit Vps26. Cells were infected and analyzed as in **C**. In all siRNA knockdown experiments, the % knockdown of the indicated protein as determined by the relative band intensity in the western is indicated below the blot panel.

The implication of putative clathrin adaptor sites in Vpu-mediated tetherin antagonism led us to test whether inhibition of clathrin function inhibits Vpu activity. Overexpression of the C-terminal fragment of the neuronal adaptor AP180 (AP180c) that inhibits clathrin/membrane interactions [42], which was recently shown to inhibit tetherin downregulation from the surface [43], specifically blocked Vpu-dependent particle release of HIV-1 wt from 293T cells expressing tetherin (Figure 6A), indicating clathrin-dependent subcellular trafficking is essential for Vpu activity. Infectious yield could not be determined in this experiment because AP180c overexpression inhibits envelope processing and blocks clathrin incorporation into particles, which has been shown to play a role in retroviral particle infectivity [44], [45]. Interestingly AP180c expression, like UBAP1 siRNA treatment, enhanced total Vpu expression levels, indicating clathrin-dependent transport is involved in the turnover of Vpu. Visualization of Vpu-YFP localization in 293T/tetherin cells overexpressing AP180c showed vesicular rather than peri-nuclear localization similar to that seen in the same cells infected with HIV-1 Vpu ELV (Figure 6B), and this was not apparent in the parental (tetherin negative) 293T cells, suggesting again this difference was driven by interaction with tetherin.

The similarities between the EXXXLV motif and acidic dileucine sorting signals, and its functional replacement with the promiscuous ENTSLL motif in HIV-1 Nef led us to test whether the major known adaptors involved in trafficking of membrane proteins between the PM, endosomes and Golgi compartments were required for Vpu-mediated tetherin antagonism. Clathrin-mediated endocytosis requires AP-2, whereas AP-3 controls early-to-late endosomal/lysosomal trafficking. AP-1 plays a role in trafficking of cargo between early endosomes and the TGN, with evidence that it can function in either direction [32]. To this end we examined the effects of siRNA-mediated silencing of AP-1 (AP-1γ1), AP-2 (AP-2μ1), AP-3 (AP-3μ1/AP-3δ1). Depletion of AP-2 by RNAi in 293T/tetherin cells had only minor effects on Vpu-dependent virus particle release, suggesting that unlike SIV Nef and HIV-2 Env, and consistent with the reports that Vpu does not enhance tetherin endocytosis, AP-2 activity is dispensable for Vpu-mediated tetherin antagonism (Figure 6C). Similarly AP-3μ1/AP-3δ1 depletion had no detectable effect on tetherin activity or Vpu-mediated counteraction (Figure 6D). Furthermore, human tetherin could also be downregulated from the surface of mouse fibroblasts defective in AP-3δ1 when transduced to express Vpu [46] (Figure S3A). For AP-1, siRNA-mediated silencing was inefficient in 293T (not shown). We therefore constructed a HeLa cell line containing a doxycycline-inducible shRNA hairpin against AP-1γ1. Induction of this hairpin coupled with simultaneous depletion of AP-1γ1 by oligonucleotide transfection led to approximately 95% knockdown efficiency. This treatment had no specific effect on Vpu-dependent virus particle yield in HeLa cells indicating that tetherin-antagonism again was not compromised (Figure 6E), and furthermore tetherin surface downregulation was not defective in mouse AP-1γ1a −/− fibroblasts (Figure S3B) [47].

Finally, we examined whether there was any role for the retromer complex, in Vpu-mediated tetherin antagonism. Retromer regulates retrieval and recycling of endosomal proteins to the TGN and is known to act co-operatively or antagonistically with AP-1 (reviewed in [48]). The retromer complex consists of several sorting nexins (SNX), and a core complex containing cargo binding component, Vps34, and two essential co-factors, Vps26 and Vps29. We performed siRNA-mediated knockdown of Vps26 (Figure 6F). At levels of knockdown that were sufficient to relocalize the CD8-cation-independent mannose-6-phosphate receptor (CD8-CI-M6PR) (Figure S3C), disruption of retromer had no detectable effect of Vpu-mediated HIV-1 release from 293T/tetherin cells.

Taken together these data demonstrate that neither depletion of individual cellular adaptor proteins known to bind to (D/E)XXXL(L/I) motifs, nor disruption of retromer-mediated endosome-to-TGN retrieval, were sufficient to recapitulate the phenotype of the Vpu ELV mutant.

### Residual function of Vpu ELV requires an intact recycling signal in tetherin

**Figure 7:**
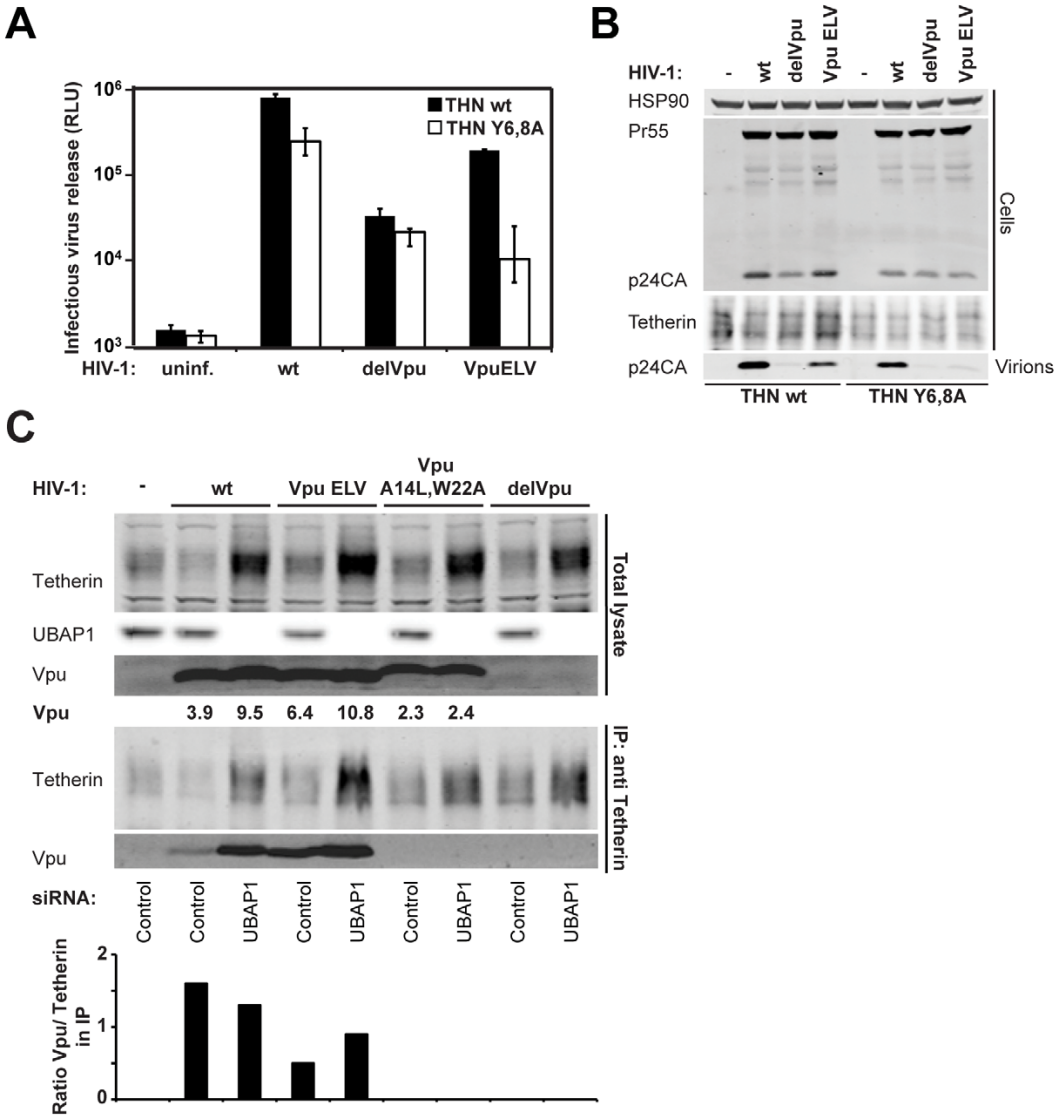
Residual activity of Vpu ELV requires an intact recycling signal in tetherin. (**A**) 293T/tetherin or 293T/tetherin Y6,8A mutant were infected with VSV-G-pseudotyped HIV-1 wt, HIV-1 delVpu or HIV Vpu ELV at an MOI of 0.5. Supernatants and cell lysates were harvested 48 h later and analyzed for infectious virus release on HeLa-TZMbl as in Figure 1C. (**B**) Corresponding Western blots of cell lysates and virions from **A**. (**C**) 293T/tetherin Y6,8A cells treated with control or UBAP1 specific siRNAs were infected with VSV-G-pseudotyped HIV-1 wt, HIV Vpu ELV, HIV-1 Vpu A14L/W22A or HIV-1 delVpu at an MOI of 2. 48 h post infection, cells were lysed and immunoprecipitated with an anti-tetherin antibody. Lysates and immunoprecipitates were subjected to SDS-PAGE and analyzed by Western Blotting for tetherin, UBAP1 and Vpu, and analyzed by LiCor quantitative imager. Ratios of Vpu/tetherin band intensities in the coIP are plotted on the histogram below.

Recent data suggests that Vpu blocks both the transit of *de novo* synthesized tetherin to the cell surface as well as the recycling of tetherin endocytosed from the plasma membrane, with the relative importance of these processes currently a matter of debate. Tetherin recycling requires a dual tyrosine YXYXXV motif in its cytoplasmic tail that acts as a binding site for AP-2 (for internalization) and AP-1 (for recycling via the Golgi) [8], [9]. Mutation of this site enhances tetherin's surface expression, but has minor effects on its ability to restrict virus release or its sensitivity to Vpu [11], [13]. Given that the EXXXLV motif was defective for post-binding inactivation of tetherin, but retained a low residual activity against tetherin in transient transfection assays, we asked whether Vpu ELV was differentially defective against tetherin mutants bearing lesions in its own sorting sequence. We infected 293T/tetherin and 293T/tetherin Y6,8A cells with HIV-1 wt, HIV-1 delVpu and HIV-1 Vpu ELV at fixed dose (MOI 1) and measured viral release 48 h later (Figure 7A and B). Vpu-defective viral release was approximately 35-fold reduced from 293T/tetherin cells compared to the wildtype virus, and as expected NL4.3 Vpu ELV had an intermediate phenotype in this assay (6 fold less release than wt). However, in 293T/tetherin Y6,8A all residual antagonistic activity of Vpu ELV was abolished with viral release equivalent to that of the Vpu-deleted virus. By contrast the wildtype virus retained the majority of its anti-tetherin activity. This again was not due to a defect of Vpu interaction with tetherin, as immunoprecipitation of tetherin after UBAP1 siRNA treatment demonstrated equivalent levels of Vpu and Vpu ELV co-precipitation from both tetherin and tetherin Y6,8A expressing cells (Figure 7C). Thus residual activity of Vpu ELV requires that tetherin retains its capacity to recycle from the PM. These data suggest that Vpu ELV is specifically defective in blocking tetherin transit to the PM, implying that Vpu/tetherin complexes are re-routed in a Golgi-associated compartment into a pathway that ultimately results in tetherin's endosomal destruction. In the absence of the EXXXLV sequence, tetherin/Vpu ELV complexes traffic to the PM. The residual activity of Vpu ELV therefore may be reflective of steric inhibition of tetherin function.

The dominant-negative mutant of dynamin 2 (K44A) has also been described to have an intermediate effect on tetherin counteraction by Vpu, compared to the complete disruption of HIV-2 Env function which, like SIV Nef, is dependent on AP-2 and endocytosis [43]. We transfected increasing doses of HA-tagged dominant negative dynamin 2 or the wildtype protein along with HIV-1 proviruses into 293T/tetherin and parental cells (Figure S4A). In agreement with Lau et al [43] dominant negative dynamin 2, but not equivalent levels of the wild type dynamin 2 partially blocked the release of wildtype HIV-1 (Figure S4A–D). Furthermore dominant negative dynamin 2 expression levels in these assays were sufficient to block transferrin uptake in parallel cultures (Figure S4B). Interestingly dominant negative dynamin 2 also blocked residual Vpu ELV-mediated, and even the low level Vpu-defective viral release proportionally (7X, 4X and 8X for WT, Vpu-defective or Vpu ELV respectively), suggesting that this effect was independent of the ELV motif. Given that dominant negative dynamin 2 inhibits tetherin endocytosis [43], this data suggests that its effect on restriction may be due more to the build up of tetherin at the cell surface that cannot be turned over rather than a direct effect on Vpu function itself.

### Vpu ELV is defective for tetherin antagonism in CD4+ T cells after treatment with type-1 interferon

**Figure 8:**
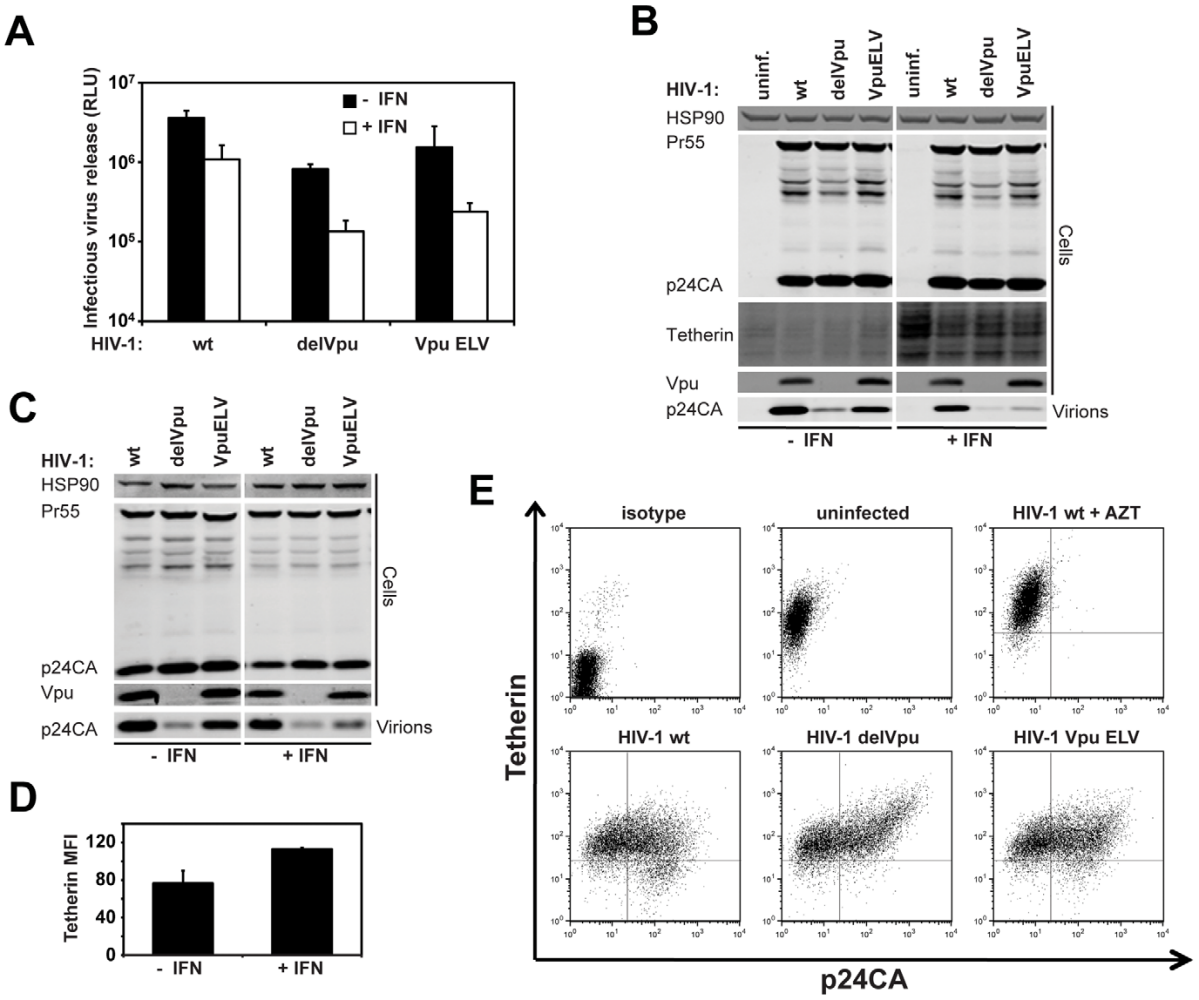
The EXXXLV motif is essential to counteract tetherin-mediated restriction of cell-free HIV-1 particle release from CD4+ T cells treated with type-1 interferon. Jurkat cells were infected with the indicated HIV-1 mutant at an MOI of 1. 16 h later the cells were treated or not with 5000 U/ml universal type-I interferon. Cell lysates and viral supernatants were harvested a further 24 h later and analyzed for infectivity on HeLa-TZM (**A**) or physical particle yield and cellular viral and tetherin expression by quantitative Western blotting (**B**). (**C**) A representative example of primary human CD4+ T cells treated as in (**B**) and the MFI of surface tetherin levels on these cells with or without 24 h type-I interferon treatment as analyzed by flow cytometry (**D**). (**E**) Human CD4+ T cells were infected with the indicated virus. 48 h later cells were stained for surface tetherin and intracellular p24CA and analyzed by flow cytometry.

The results presented hitherto have demonstrated a requirement for the Vpu ELV motif in counteracting tetherin in cells stably expressing it or mutants thereof, and that its is required in constitutively-expressing target cells such as Jurkat to reduce surface tetherin levels. However some studies have cast doubt as to whether tetherin degradation and/or surface reduction is essential for Vpu function. Furthermore, in contrast to 293T/tetherin, release of HIV-1 Vpu ELV from HeLa was only 3-fold less efficient than wildtype in one round release (Figure S5). We therefore explored whether there was a requirement for the ELV motif in HIV-1 release from physiologically relevant target cells, namely Jurkat or primary human CD4+ T cells, particularly after treatment with type-1 interferon. Infection of Jurkat at an MOI of 1 resulted in a partial defect in release of the HIV-1 Vpu ELV mutant compared to the controls (Figure 8A and B). However induction of higher tetherin expression by overnight treatment with universal type-1 interferon effectively reduced HIV-1 Vpu ELV particle release to levels similar to that of the Vpu-defective control while only reducing the wildtype release moderately. Similarly interferon treatment of purified activated human CD4+ T cells led to a selective defect in the production of cell-free HIV-1 Vpu ELV virions (Figure 8C) consistent with a concomitant upregulation of surface tetherin levels (Figure 8D), and surface tetherin downregulation (Figure 8E). Taken together with results presented above, these data demonstrate that the ability to downregulate and degrade tetherin imparted by the EXXXLV motif is required for cell-free virion release from relevant primary HIV target cells, and becomes essential when tetherin expression is enhanced by an antiviral stimulus.

## Discussion

In this study we have identified a determinant within the second alpha helix of the cytoplasmic tail of HIV-1 NL4.3 Vpu, E_59_XXXL_63_V_64_, which is required for efficient antagonism of tetherin. Mutation of this site blocks the ability of Vpu to mediate tetherin downregulation from the cell surface and its ESCRT-dependent degradation, but does not abolish its interaction with tetherin, nor recruitment of β-TrCP2 or the ESCRT-0 component HRS. Importantly, this motif is required to counteract tetherin in CD4+ T cells, particularly after their exposure to type-I interferon. Vpu ELV mutants localize to the cell surface and early/recycling endosomal compartments rather than the TGN by virtue of their interaction with tetherin. This is consistent with a role for this determinant in Vpu-mediated inhibition of the transit of newly synthesized and/or recycling tetherin to the cell surface, its internal sequestration and targeting for endo-lysosomal degradation. However, while we could functionally complement Vpu function by grafting the D/EXXX(L/I/M) motif from HIV-1 Nef in place of helix-2, we could detect no effect of depletion of the canonical clathrin adaptor proteins AP-1, AP-2 or AP-3 on Vpu-mediated tetherin antagonism. The inhibition by AP180c overexpression, however, implicates clathrin function in Vpu-mediated tetherin antagonism. Finally, residual Vpu ELV activity against tetherin was entirely dependent on an intact recycling motif in tetherin's cytoplasmic tail, suggesting that this motif differentially affects antagonism of newly synthesized tetherin rather than pre-existing pools recycling to the PM. These results are in contrast to the recent study by Lau et al who were unable to demonstrate a phenotype for an LV63,64AA mutant despite interfering with Vpu activity with AP180c [43]. Given that we have demonstrated phenotypes in several cellular systems including CD4+ T cells for the Vpu ELV mutant, the reason for this discrepancy is unclear.

There has been much debate as to whether the reduction of tetherin levels at the plasma membrane is required to counteract its antiviral activity, particularly in CD4+ T cells (reviewed in [10]). In our hands, tetherin surface levels are reduced in HIV-1 infected primary T cells and Jurkat cells. However, under these conditions, the viral release phenotype of the Vpu ELV mutant is only a few-fold different than the wildtype protein. Interestingly this changes upon treatment of the cells with type-I interferon, which upregulates tetherin expression levels. In this case Vpu ELV mutant release is reduced almost to that of the Vpu-defective virus, but only minor further reductions are observed for the wildtype virus. This therefore indicates that the requirement for surface reduction/degradation of tetherin becomes much more important at higher expression levels of the restriction factor (something that has been suggested previously by Goffinet et al [49]). Recent conflicting data have addressed the effect of tetherin and interferon on cell-cell transmission between T cells [50], [51], which when taken together show that if tetherin does restrict this mode of virion transfer, it is far less efficient than its effect on cell-free viral release. Thus, the fact that tetherin antagonism is such a highly conserved attribute amongst primate immunodeficiency viruses implies its importance in vivo. Since interferon treatment of CD4+ T cells magnifies the defective phenotype of a Vpu ELV mutant, the ability to mediate tetherin's surface reduction and target it for endosomal degradation is likely to be essential for the virus to avoid restriction under proinflammatory conditions it is likely to encounter in vivo, particularly during acute infection [52], [53].

Our results are consistent with the notion that Vpu blocks delivery of tetherin to the plasma membrane [13], [54] and suggest that Vpu exerts its effect on tetherin trafficking in the TGN/recycling compartment in a manner determined in part by this putative sorting sequence. It is now clear that Vpu-mediated tetherin degradation is ubiquitin-dependent and occurs in lysosomes rather than early reports of proteasomal processing [15], [16], and that this process requires the ESCRT pathway [19]. Depletion of both TSG101 and Vps4 inhibits Vpu-mediated tetherin degradation, and recruitment of HRS (ESCRT-0) has been reported to be required for Vpu-mediated tetherin antagonism [19], as has ubiquitination on multiple residues in the tetherin cytoplasmic tail [18]. However tetherin's ultimate degradation itself is not essential for Vpu or other lentiviral countermeasures to inactivate it. Our recent observations with the novel ESCRT-I subunit, UBAP1, ([22] and the results presented herein), demonstrate that ESCRT-I function is unlikely to be required for tetherin antagonism, but its commitment to endosomal degradation is. Interestingly, the concomitant enhancement of wildtype Vpu levels, both co-precipitating with tetherin and at steady state, suggest that Vpu is likely co-degraded with its target.

**Figure 9:**
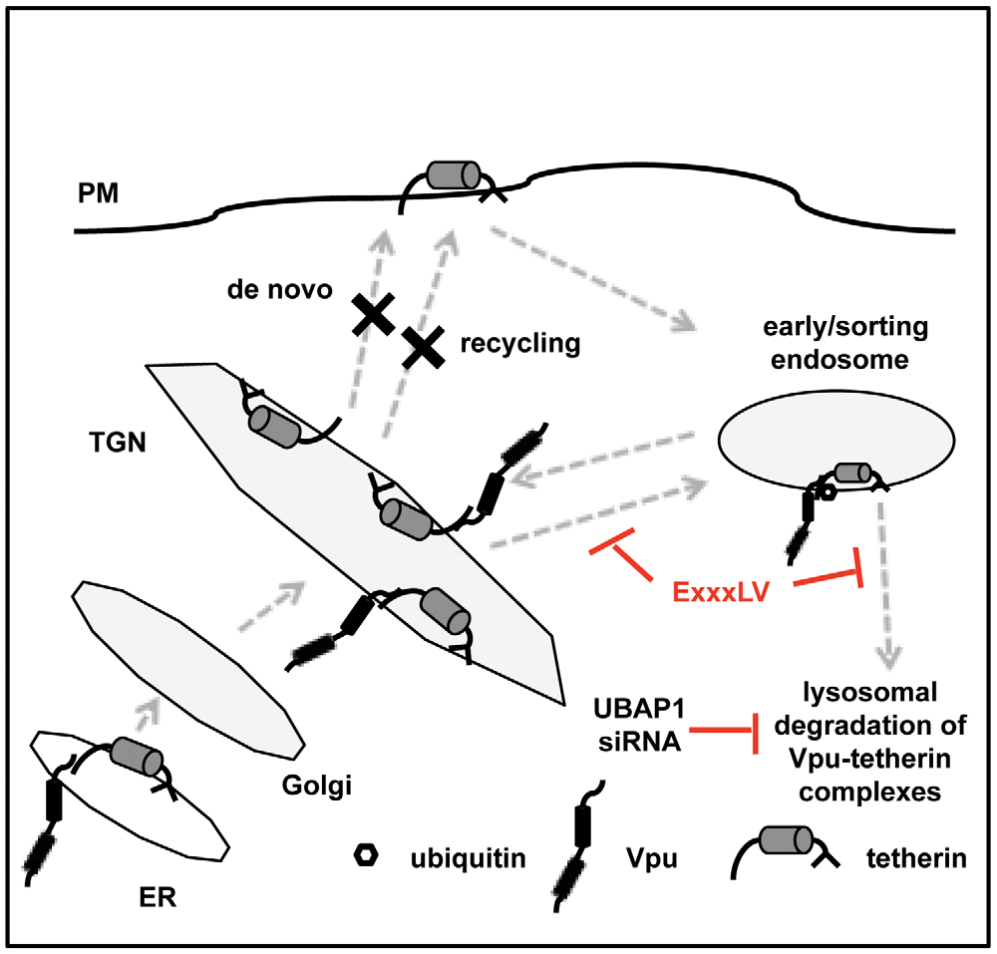
Model for the role of the EXXXLV motif in tetherin antagonism. Tetherin is expressed at the plasma membrane where it can become incorporated into viral particles or recycles constitutively via early/sorting endosomal compartments and the TGN. Vpu interacts with tetherin in the TGN (and perhaps earlier) through TM-domain-mediated interactions. In the presence of a functional EXXXLV motif, tetherin/Vpu complexes are prevented from trafficking to the PM and routed for ESCRT-dependent endosomal degradation via a clathrin-dependent mechanism. In the absence of an EXXXLV motif, tetherin/Vpu complexes recycle via the PM dependent on the YXYXXV sorting sequence in the tetherin cytoplasmic tail, which interacts with AP-2 and AP-1. During the recycling process, physical interaction of Vpu and/or modification by ubiquitin ligases, such as SCF-β-TrCP2, may further interfere with tetherin function to a variable degree in the absence of cell-surface downregulation.

Alongside the current literature our data suggests that Vpu interaction with tetherin leads to a differential trafficking of tetherin in the TGN and/or recycling compartments rather than enhancing tetherin internalization (Figure 9). This inhibits forward trafficking of either recycling or newly synthesized tetherin to the PM, and commits it to a pathway that ultimately targets it to endolysosomal compartments for degradation. We suggest that it is this commitment, regulated by the EXXXLV motif in second helix, rather than degradation *per se*, that is principally responsible for antagonizing tetherin. Where Vpu interacts with tetherin in the cell is likely to be related temporally to the viral replication cycle, tetherin expression level, and its natural turnover rate. Vpu is expressed “late” in replication from the same mRNA as Env [55], at the time new virions are being built. Thus Vpu must deal with two pools of tetherin; pre-existing protein in the periphery recycling via the TGN [9], and *de novo* synthesized tetherin trafficking through the Golgi *en route* from the endoplasmic reticulum (ER) to the surface. Therefore we predict that Vpu must interact with tetherin in TGN-associated compartments to engage these two pools of tetherin, although our recent data suggests that binding to newly synthesized tetherin may occur prior to this in the ER [14]. Thereafter, a clathrin-dependent sorting event determined by the helix-2 EXXXLV motif precludes Vpu/tetherin complexes transiting to the PM. This intracellular sequestration of tetherin is further coupled to late endosomal targeting and ESCRT-dependent degradation through the recruitment of HRS and tetherin ubiquitination [18], [19]. In line with this, global disruption of early to late endosomal transition by dominant negative Rab7a also appears to inhibit Vpu activity [23]. Interestingly, ubiquitinated cargo destined for ESCRT-dependent degradation via HRS recruitment has been shown to partition differentially to areas of early/sorting endosomal membranes rich in flat clathrin lattices, thereby anchoring it away from the recycling machinery [56]. Recruitment of tetherin into such structures in the sorting compartment may be sufficient to antagonize its function, offering an explanation as to why clathrin is essential for Vpu activity but dynamin 2 mutants only effect the residual activity of Vpu ELV. Because Vpu activity was insensitive to retromer (Vps26) depletion, which is essential for endosome-to-TGN retrieval and recycling [48], we suggest that Vpu targets tetherin into this pathway from the TGN to endosomal compartments, and an ensuing swift degradation accounts for the observed sequestration in TGN46 positive compartments. In the absence of the EXXXLV motif, tetherin is not committed to this differential sorting in the TGN, and Vpu/tetherin complexes are targeted to the cell surface and thereafter back into the recycling system, accounting for the localization of Vpu ELV in infected cells and its incorporation into virions. Therefore in addition to acting as an adaptor for the recruitment of ESCRT-0 and E3 ubiquitin ligase activity to tetherin, we propose that through the EXXXLV motif, Vpu directly chaperones associated tetherin molecules into an endosomal compartment from which they cannot recycle to the PM.

EXXXLV mutants retain some residual antagonistic activity that is entirely dependent on the AP-1/AP-2-binding motif in tetherin. This would suggest that the action of the EXXXLV motif does not solely account for the inhibition of tetherin by Vpu. Vpu ELV still interacts with β-TrCP2, suggesting that tetherin ubiquitination, which is important for counteracting its activity [18], may still take place during the recycling process and effect antiviral function. Also physical interaction between Vpu and tetherin may be sufficient to interfere with some level tetherin function provided tetherin can still associate with the clathrin-dependent endocytic machinery. Since biochemical evidence strongly favors a direct cross-linking model for tetherin's antiviral activity [7], either of these processes could in principle affect the qualitative nature of tetherin distribution at viral assembly domains at the PM such that tethers do not form efficiently, something that is plausible in the light of Habermann et al's quantitative Cryo-EM analysis of tetherin localization at the PM in infected HeLa cells [57]. The relative efficiency of this “secondary” inhibition of tetherin activity will thus be dependent on cellular tetherin levels and the temporal stage of viral replication (ie: the expression level of Vpu). Since Vpu-defective mutants of HIV-1 in some studies show enhanced cell-to-cell transmission and that tetherin may play a role in virological synapse formation [51], [58], [59], such a differential effect on *de novo* synthesized versus the recycling pool tetherin may favor viral transmission during early stages of viral production, but effectively antagonize induction of tetherin expression and potent restriction by a pro-inflammatory response.

We have so far been unable to demonstrate direct interaction between the Vpu EXXXLV motif and adaptors AP-1, 2 or 3 that are known to bind acidic dileucine signals via a hemicomplex of their sigma and adaptin subunits [32]. These interactions have been demonstrated in yeast 3-hybrid assays previously for HIV-1 Nef [36], [37], but not all interactions are amenable to this method and in our hands the Vpu cytoplasmic tail is a constitutive activator of transgene expression precluding its use. Furthermore, the low affinity of these interactions, their transient nature, and the complex interactions of the adaptor protein with membrane lipids means that adaptor binding is rarely measurable in co-precipitations from cells. The context dependency of (D/E)XXXL(L/I) motifs that governs which adaptor they bind to *in vivo* is poorly defined at present, meaning that RNAi-depletion is the most reliable method for identifying the cellular factor involved. While individual depletion of AP-1, 2 or 3 in our experiments had no effect on Vpu-mediated tetherin antagonism, replacement of the second alpha helix with the promiscuous AP-binding peptide from HIV-1 Nef did recover function. This site in Nef is essential for several functions including CD4 and MHC Class I downregulation, the former being AP-2-dependent [37], the latter requiring AP-1 [38]. Importantly, binding of AP-2 to this site in SIV Nef proteins is required for their counteraction of non-human primate tetherins [26]. Thus there are several possibilities. The EXXXLV motif in Vpu might be a promiscuous AP-binding site and adaptor usage may be redundant. The subcellular localization of tetherin/Vpu ELV complexes in EEA1 positive endosomes and the partial effect of dominant negative dynamin 2 would argue against a significant role for AP-2, the major regulator of clathrin-mediated transport from the PM. However toxicity associated with simultaneous knockdown of multiple adaptors has precluded us from addressing this possibility so far. Alternatively, replacement of the second alpha with the Nef peptide recovers function because it confers AP-2 binding to Vpu, thus allowing Vpu to counteract tetherin in a manner similar to SIV Nef and HIV-2/SIV Envs that both require AP-2 interactions [24]–[26], [60]. However, these data do underscore that linking Vpu to the clathrin trafficking machinery promotes its ability to counteract tetherin.

If Vpu-mediated tetherin antagonism is clathrin-dependent, but independent of AP-1, AP-2 or AP-3 (for which the importance of clathrin is debated), what other adaptors might be important? Two further heterotetrameric adaptors, AP-4 and AP-5, have been identified, but understanding of their role in subcellular trafficking is limited and at present they are not known to bind to acidic dileucine motifs or to control clathrin-mediated transport [32], [61]. The monomeric GGA (Golgi-associated, γ-ear containing, ARF-cofactors) 1–3 proteins that also function as clathrin adaptors that regulate Golgi-to-endosome transport are potentially attractive candidates, whose dysregulation has been reported to inhibit HIV-1 assembly [62]. However, known GGA-binding motifs in cellular cargoes correspond to a DXXLL consensus where the spacing of the leucines from the acidic residue is thought critical.

The (E/D)XXXL(V/M/I) motif is conserved in Vpu proteins from HIV-1 M group subtypes A, B, D, G and H (Figure S2), as well as in Group O (www.hiv.lanl.gov). While Group O Vpus cannot antagonize tetherin [63], this maps to defective TM domain-mediated interaction and the membrane proximal hinge region. When replaced by those from a Group M Vpu these are sufficient to confer tetherin inactivation implying that C-terminal determinants retain function [64]. In Group M clades C and F, the equivalent position is (D/E)XXXL(S/A) respectively, suggesting that the site may not be functional in those Vpus, with the caveat that the second position in the dileucine motif is less important. Intriguingly both these subtypes bear EYXXL(L/I) motifs in the membrane proximal region of their cytoplasmic tails that encompass both a putative tyrosine and dileucine-based sorting sequence. Evidence from Ruiz et al [34] has shown that a model subtype C Vpu localizes to the PM rather than the TGN and that this is in part due to this motif. While LL mutations in this subtype C Vpu confer T-cell line replication phenotypes suggestive of a failure to downmodulate tetherin, no direct experiments on the role of this motif in tetherin-mediated HIV-1 release have been thus far performed, nor whether this site can bind known clathrin adaptors.

In summary our data implicate a trafficking determinant in the Vpu cytoplasmic tail that is required for tetherin downregulation, degradation and efficient antagonism, and suggest that it governs differential sorting of Vpu/tetherin complexes in the TGN to prevent forward transit of tetherin to the PM and viral budding sites.

## Materials and Methods

### Cells and plasmids

HEK293T, HeLa and Jurkat cells were obtained from ATCC (American Tissue Culture Collection). 293T/tetherin and 293T/tetherin-delGPI and HT1080/tetherin-HA are cell lines stably expressing human tetherin or mutant thereof, with or without a hemagglutinin (HA) epitope tag inserted at nucleotide 463, which has been previously described [1], [65]. The reporter cell line HeLa-TZMbl, was kindly provided by John Kappes through the NIH AIDS Reagents Repository Program (ARRP). All adherent cells were maintained in Dulbecco's modified Eagle medium (DMEM) (Invitrogen, UK) supplemented with 10% fetal calf serum and Gentamycin; T-cell lines were grown in Roswell Park Memorial Institute medium (RPMI) supplemented with 10% fetal calf serum and Gentamycin. Murine fibroblasts from *pearl* and AP1γ1a deficient mice and derivative in which AP3μ or AP1γ1a were re-expressed were kindly provided by Andrew Peden [46] and Peter Schu [47] respectively. These cells were transduced to express HA-tagged human tetherin using pLHCX-THN-HA463 [65] and maintained in hygromycin selection.

Wildtype HIV-1 NL4.3 (obtained from NIH-ARRP), a Vpu-defective counterpart and pCR3.1 Vpu-HA containing a modified codon optimized NL4.3 Vpu has been described previously [31]. All second alpha helix mutants of Vpu and mutations in the NL4.3 proviral genome were generated by Quick-change site-directed mutagenesis PCR according to standard protocols using Phusion-II polymerase (New England Biolabs). Vpu-Nef chimeras and corresponding mutants were made with long reverse PCR primers encoding Nef clathrin adaptor binding sites, cloned into pCR3.1 expression vectors encoding tagged or untagged tetherins which have been described elsewhere [1]. β-TrCP2 was cloned from HeLa cDNA and inserted into pCR3.1 with a C-terminal myc-tag. pCR3.1 HA-HRS was kindly provided by Juan Martin-Serrano [66]. pCR3.1 dynamin 2-HA and dominant negative dynamin 2-HA have been previously described by [31].

Primary human CD4+ T cells were isolated from fresh venous blood drawn from healthy volunteers. CD4+ T cells were purified from total peripheral blood mononuclear cells (PBMC) isolated by lymphoprep (AXIS-SHIELD) gradient centrifugation using a CD4+ T cell Dynabeads isolation kit (Invitrogen). T cells were then activated for 48 h using anti-CD3/anti-CD28 magnetic beads (Invitrogen). The beads were then removed cells were then maintained in rhIL-2 (20 U/ml) (Roche).

### Production of viral and vector stocks

For full-length HIV-1 stocks pseudotyped with the Vesicular Stomatitis Virus Glycoprotein (VSV-G), 293T cells were transfected with 2 µg of proviral plasmid and 200 ng of pCMV VSV-G. 48 h post-transfection, viral stocks were harvested and endpoint titers were determined on HeLa-TZMbl cells as described below [25].

### Virus release assay

For transient-transfection-based virus release assays, subconfluent 293T cells were plated on 24 well plates and transfected with 500 ng proviral clone, in combination with 50 ng of tetherin and 25 ng of Vpu-HA or mutants using 1 µg/ml polyethyleneimine (Polysciences). The medium was replaced 5 h and 16 h post-transfection, cells were harvested after 48 h. The infectivity of viral supernatants was determined by infecting HeLa-TZMbl, 48 h later cells were assayed for β-galactosidase activity using the chemiluminescence Tropix GalactoStar kit (Applied Biosystems). For biochemical analysis of virus particle release, supernatants were filtered (0.22 µm) and pelleted through a 20% sucrose/PBS cushion at 20,000 g for 90 min at 4°C, and pellets were lysed in SDS-PAGE loading buffer. Virion and cell lysates were then subjected to SDS-PAGE and Western blotted for HIV-1 p24CA (monoclonal antibody 183-H12-5C; kindly provided by B Chesebro through the NIH ARRP), rabbit anti-Hsp90 (Santa Cruz Biotechnologies), monoclonal mouse anti-HA.11 (Covance), polyclonal rabbit anti-HA (Rockland) and/or Vpu (rabbit polyclonal; kindly provided by K. Strebel through the NIH ARRP [67], and visualized by LiCor apparatus using fluorophores conjugated secondary antibodies (IRDye 800 Goat anti-rabbit, IRDye 680 Goat anti-mouse).

### One round viral release assay

5×10^5^ cells (293T/tetherin, Jurkat or CD4+ T cells) were infected with VSV-G-pseudotyped HIV-1 wt, HIV-1 delVpu or HIV-1 Vpu ELV at an MOI of 0.5–1. 16 h post infection medium was replaced and cells (treated or not with 5000 U/ml of universal type-1 interferon (PBL InterferonSource)) were cultured for a further 24 h. The cells harvested, the infectivity of viral supernatants was determined by infecting HeLa-TZMbl and biochemical analysis of virus particle release was performed as in Virus release assay. For examining tetherin degradation HT1080 cells stably expressing tetherin-HA were infected with VSV-G-pseudotyped HIV-1 wt, HIV-1 delVpu or HIV-1 Vpu ELV virus stocks at a multiplicity of infection (MOI) of 2 to ensure that approximately 90% of the cells were infected. The medium was replaced 4 h after infection. 48 h post infection cell lysates were harvested and processed as described above.

### Flow cytometry

HeLa cells were transfected with 400 ng of pCR3.1 GFP and 400 ng of pCR3.1 Vpu-HA or indicated mutants. 48 h post transfection the cells were harvested and stained for surface tetherin using a specific anti-BST2 monoclonal IgG2a antibody (Abnova) and goat-anti-mouse IgG2a-Alexa633 conjugated secondary antibody (Molecular Probes, Invitrogen, UK). Tetherin expression on GFP positive cells was then analyzed using a FacsCalibur flow-cytometer (Becton Dickinson) and the FlowJo software. Murine fibroblasts were transduced with the pMigR1-based retroviral vector pCMS28-IRES-eGFP or a derivative expressing NL4.3 Vpu. 48 h after transduction the cells were stained for surface HA versus GFP expression. Jurkat or CD4+ T cells were infected with VSV-G-pseudotyped HIV-1 wt, HIV-1 delVpu or HIV-1 Vpu ELV at an MOI of 1. 48 h post infection cells were stained for surface tetherin expression as above, then fixed and permeabilized for 20 minutes (Cytofix/cytoperm Fixation/Permeabilization kit, BD Biosciences) and stained for intracellular HIV-1 p24CA using the KC57 antibody conjugated to PE (Beckman- Coulter).

### Immunofluorescence microscopy

Cells were grown on coverslips and infected with VSV-G-pseudotyped HIV-1 wt or HIV-1 Vpu ELV, 48 h later cells were fixed in 4% paraformaldehyde/PBS, washed with 10 mM glycine/PBS, and permeabilized in 1% bovine serum albumin/0.1% Tritin-X100/PBS for 15 min. The infected cells were stained using anti-rabbit polyclonal Vpu in combination with sheep anti-human TGN46 (AbD Serotec), mouse anti-EEA1 (BD Biosciences), mouse anti-CD63 (Developmental Studies Hybridoma Bank, University of Iowa) or mouse polyclonal anti-BST-2 (Abnova) followed by the appropriate secondary antibodies conjugated to Alexa 488 or 594 fluorophores (Molecular Probes, Invitrogen). The cells were then mounted on glass slides using ProLong AntiFade- 4′,6-diamidino-2-phenylindole (DAPI) mounting solution (Molecular Probes, Invitrogen). Cells were visualized with a Leica DM-IRE2 confocal microscope. Images were analyzed using Leica Confocal Software and ImageJ.

### Immunoprecipitations

293T cells stably expressing tetherin were transfected twice over 48 h with siRNA oligonucleotide against UBAP1 targeting CTCGACTATCTCTTTGCACAT or Non-targeting siRNA was used as control (Dharmacon). The cells were then infected with VSV-G-pseudotyped HIV-1 wt, HIV-1 delVpu, HIV-1 Vpu ELV or HIV-1 Vpu A14L,W22A at an MOI of 2. 48 h post infection the cells were lysed on ice for 30 min in buffer containing 50 mM Tris-HCL pH 7.4, 150 mM NaCl, complete protease inhibitors (Roche) and 1% digitonin (Calbiochem). After removal of the nuclei, the supernatants were immunoprecipitated with 5 µg/ml mouse monoclonal anti-BST2 antibody (eBiosciences) for 1.5 h at 4°C. Sepharose-protein G beads were washed in lysis buffer before they were added to the samples and incubated for further 3 h. The beads were washed extensively in lysis buffer containing 0.1% digitonin and resuspended in SDS-PAGE loading buffer. Cell lysates and immunoprecipitates were subjected to SDS-PAGE, and Western blot assays were performed using a rabbit anti-Vpu antibody (kindly provided by K Strebel through the NIH ARRP), polyclonal rabbit anti-tetherin antibody (kindly provided by K Strebel through the NIH ARRP) and polyclonal rabbit anti-UBAP1 antibody (Proteintech), and visualized by ImageQuant using corresponding HRP-linked secondary antibodies (New England Biolabs, UK). For HRS/Vpu coIP, 293T cells were co-transfected with 700 ng of pCR3.1 HA-HRS and pCR3.1 Vpu-YFP, pCR3.1 Vpu ELV-YFP or pCR3.1 YFP expression plasmids. 48 h post transfection the cells were lysed in buffer containing 0.1 M MES-NaOH pH 6.5, 1 mM magnesium acetate, 0.5 mM EGTA, 200 µM sodium ortho-vanadate, 10 mM NEM, complete protease inhibitors (Roche) and 1% digitonin. After removal of the nuclei, the supernatants were immunoprecipitated with 5 µg/ml monoclonal mouse anti-HA.11 antibody (Covance). Immunoprecipitation was performed as described above and Western blot assays were performed using a polyclonal rabbit anti-HA antibody (Rockland) and an anti-Vpu antibody.

### Crosslinking IP

293T cells were co-transfected with 700 ng of pCR3.1 myc β-TrCP 2 and pCR3.1Vpu-HA, pCR3.1 Vpu ELV-HA, pCR3.1 Vpu 2/6A-HA or pCR3.1 YFP expression plasmids. 48 h post transfection, Crosslinking Immunoprecipitation was performed as previously described [68]. Cell lysates and immunoprecipitates were subjected to SDS-PAGE, and Western blot assays were performed using a rabbit anti-Vpu antibody and mouse anti-myc antibody (kindly provided by M. Malim), and visualized by ImageQuant (GE) using corresponding HRP-linked secondary antibodies (New England Biolabs).

### siRNA-mediated clathrin adaptor knockdown

293T cells stable expressing tetherin or HeLa cells were seeded at a density of 2×10^5^ cells per well in a 12 well plate. After 3 h, the first transfection was performed. For each well, 3 µl Oligofectamine (Invitrogen) was added to 10 µl of Opti-MEM (Life Technologies), this solution was added to 5 µl of 20 µM siRNA in 85 µl of Opti-MEM according to manufactures protocol. For AP-1 knockdown, HeLa cells stably expressing doxycycline-inducible pTRIPZ shRNA against AP-1γ1 (OpenBiosystems) were used in combination with siRNA oligonucleotide against AP-1γ1 targeting AAGAAGATAGAATTCACCTTT. For AP-2 knockdown, SMARTpool siRNA targeting the AP-2 µ1 subunit was used (Dharmacon). For AP-3 knockdown, SMARTpool siRNA targeting the AP-3 µ1 subunit was used in combination with SMARTpool siRNA targeting the AP-3δ1 subunit (Dharmacon). For Vps26 knockdown, siRNA targeting the AACCACCTATCCTGATGTTAA sequence was used (Qiagen). On Non-targeting siRNA was used as control (Dharmafect). The cells were reseeded into a 24 well plate on day 2 and a second transfection was performed according to manufactures protocol. The cells were infected 3 h post transfection with VSV-G-pseudotyped HIV-1 wt, HIV-1 delVpu at an MOI of 0.8. The infectivity of viral supernatants was determined by infecting HeLa-TZMbl as described above. Cell lysates and viral particles were subjected to SDS-PAGE, and Western blot assays were performed using a mouse monoclonal AP-1γ1 antibody (Sigma), mouse anti-AP50 (AP-2μ1) and mouse anti-AP-3δ1antibodies (BD Bioscience), polyclonal rabbit AP-3μ1 antibody (kindly provided by M.S. Robinson) and rabbit polyclonal Vps26 antibody (Abcam).

### Ethics statement

Ethical approval for the drawing of blood and preparation of leukocyte subsets from healthy donors following written informed consent was obtained through the King's College London Infectious Disease BioBank Local Research Ethics Committee (under the authority of the Southampton and South West Hampshire Research Ethics Committee – approval REC09/H0504/39), approval number SN-1/6/7/9.

## Supporting Information

Figure S1 **Effect of Vpu helix 2 mutants on surface tetherin expression in HeLa cells.** (**A**) The full panel of helix 2 alanine scan mutants performed as described for Figure 2D. (**B**) HeLa cells were infected with the indicated VSV-G pseudotyped HIV-1 mutant at an MOI of 0.5. 48 h later cells were stained for surface tetherin and intracellular p24CA. (**C**) Productively infected Jurkat cells in Figure 2E were discriminated from those acquiring p24+ matter from the inoculum by exposing them to the same dose of wildtype HIV-1 in presence of AZT.
(TIF)

Figure S2 **Vpu EXXXL(V/M/I/L) is conserved in most HIV-1 clades.** (**A**) LogoPlots of Vpu cytoplasmic tail portions encompassing the conserved phosphorylation motif (DSGNES) and helix 2 from HIV-1 subgroup M clades A,B,D,G and H generated from sequences obtained from the Los Alamos database (www.hiv.lanl.gov). (**B**) 293T cells were transfected with NL4.3 or NL4.3 delVpu proviruses in combination with tetherin and Vpu-HA, Vpu 64I-HA, Vpu 64M-HA or Vpu 64L-HA expression vectors. 48 h post transfection, cell lysates and pelleted supernatant virions were harvested and subjected to SDS-PAGE and analyzed by Western blotting for HIV-1 p24CA, Vpu-HA and Hsp90, and analyzed by LiCor quantitative imager. (**C**) HeLa cells were co-transfected with Vpu or indicated Vpu mutant and a GFP expression construct. Cell surface staining for endogenous tetherin was analyzed by flow cytometry 48 h post transfection, as in Figure 2D.
(TIF)

Figure S3 **Effects of Vpu on surface expression of human tetherin in murine fibroblasts deficient for AP3 or AP1.** Fibroblasts from *pearl* (AP3δ−/−) (**A**) or AP-1μ1A−/− mice (**B**) or their reconstituted counterparts were transduced to express human tetherin bearing an extracellular HA-tag. The cells were then transduced with retroviral vector constructs encoding Vpu linked to GFP via an IRES. 48 h later the cells were surface stained for human tetherin expression using anti-HA antibodies. (**C**) HeLa-CD8-CI-M6PR cells were treated with control or Vps26 siRNAs. 48 h later the cells were fixed and stained for CD8 (green) and TGN46 (red).
(TIF)

Figure S4 **Effects of dominant negative dynamin 2 on Vpu-mediated HIV-1 release.** 293T/tetherin cells were transfected with the indicated HIV-1 provirus and increasing doses of HA-tagged dominant negative dynamin 2 (**A**) or the wildtype protein (**B**). 48 h later, cell lysates and viral supernatants were harvested and subjected to SDS-PAGE and analyzed by Western blotting for HIV-1 p24CA, dynamin 2-HA and Hsp90, and analyzed by LiCor quantitative imager. Histograms below the blots indicate particle release efficiency compared to wildtype virus release in the absence of dynamin 2 or increasing doses of dynamin 2 expression vector. (**C**) Corresponding infectivity of viral supernatants from **A** and **B** on HeLa-TZM cells alongside those from a parallel experiment in parental 293T cells. (**D**) 293T/tetherin cells were transfected with pCR3.1 YFP with or without increasing doses of dominant negative dynamin 2. 48 h later the cells were starved in serum-free medium for 30 minutes and then treated for a further 15 minutes with 10 µg/ml Alexa-594-conjugated transferrin, before fixation and imaging.
(TIF)

Figure S5 **Effect ELV mutant on HIV-1 infectious release from HeLa cells.** HeLa cells were infected with VSV-G pseudotyped stocks of the indicated virus at an MOI of 0.5. 48 h later the cell supernatants were harvested and infectivity determined on HeLa-TZM cells.
(TIF)
