# Supplementary figures and images for: A Cytoplasmic Tail Determinant in HIV-1 Vpu Mediates Targeting of Tetherin for Endosomal Degradation and Counteracts Interferon-Induced Restriction
Source: PLoS Pathog. 2012 Mar 29;8(3):e1002609. doi: 10.1371/journal.ppat.1002609 (PMC3315493; doi:10.1371/journal.ppat.1002609)

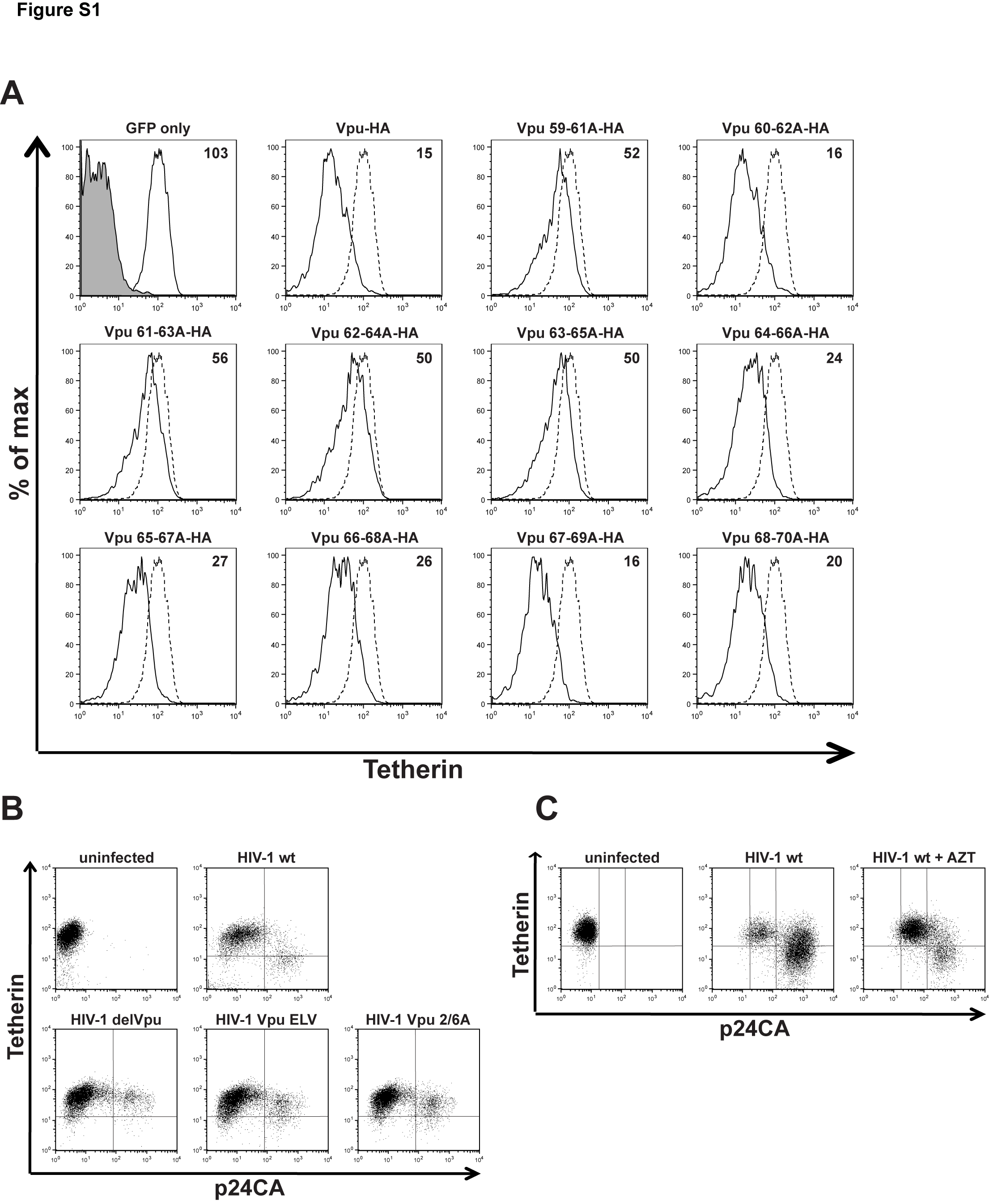

Supplement: Figure S1 — Effect of Vpu helix 2 mutants on surface tetherin expression in HeLa cells. (A) The full panel of helix 2 alanine scan mutants performed as described for Figure 2D. (B) HeLa cells were infected with the indicated VSV-G pseudotyped HIV-1 mutant at an MOI of 0.5. 48 h later cells were stained for surface tetherin and intracellular p24CA. (C) Productively infected Jurkat cells in Figure 2E were discriminated from those acquiring p24+ matter from the inoculum by exposing them to the same dose of wildtype HIV-1 in presence of AZT. (TIF) [file ppat.1002609.s001.tif]

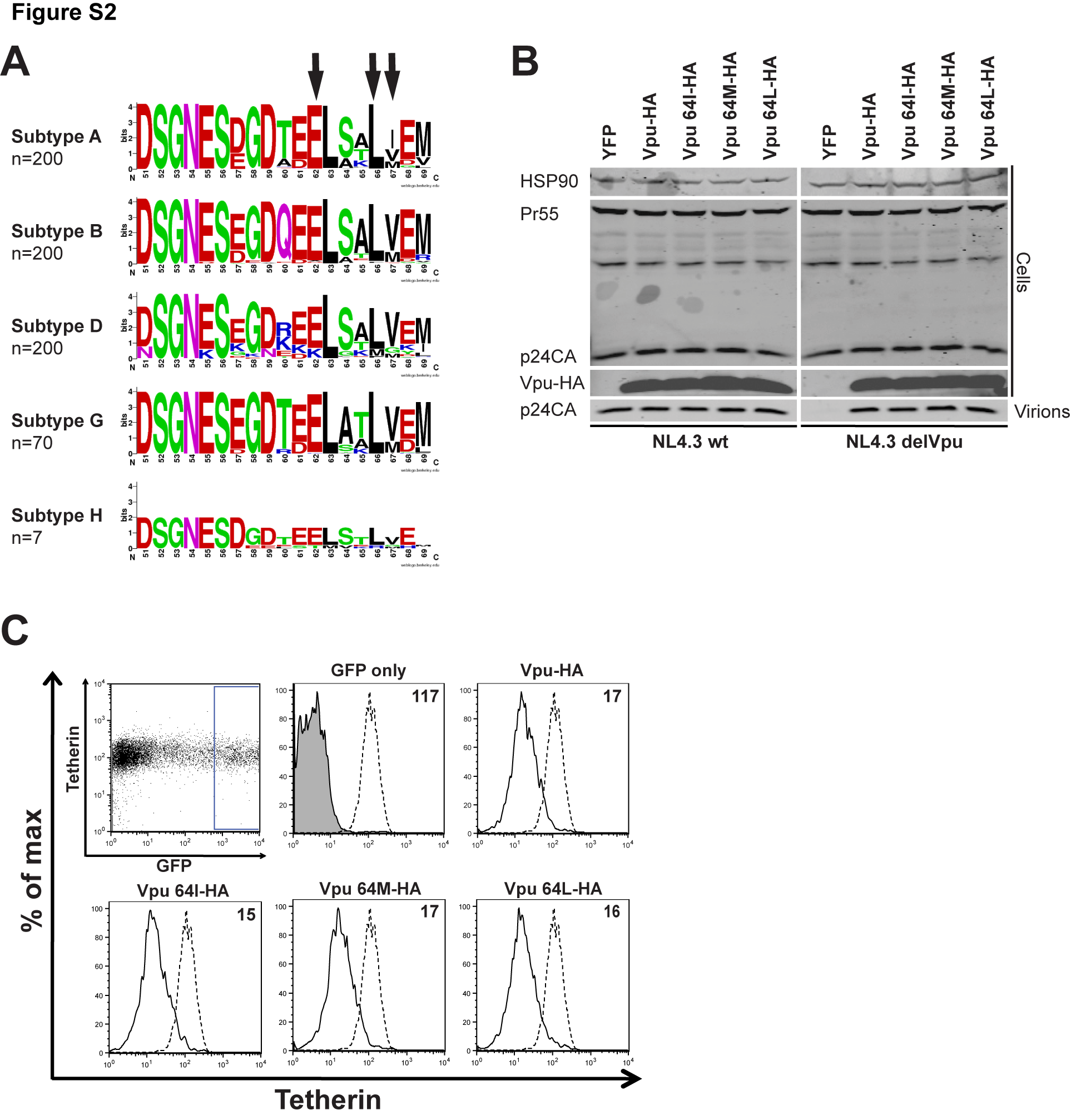

Supplement: Figure S2 — Vpu EXXXL(V/M/I/L) is conserved in most HIV-1 clades. (A) LogoPlots of Vpu cytoplasmic tail portions encompassing the conserved phosphorylation motif (DSGNES) and helix 2 from HIV-1 subgroup M clades A,B,D,G and H generated from sequences obtained from the Los Alamos database (www.hiv.lanl.gov). (B) 293T cells were transfected with NL4.3 or NL4.3 delVpu proviruses in combination with tetherin and Vpu-HA, Vpu 64I-HA, Vpu 64M-HA or Vpu 64L-HA expression vectors. 48 h post transfection, cell lysates and pelleted supernatant virions were harvested and subjected to SDS-PAGE and analyzed by Western blotting for HIV-1 p24CA, Vpu-HA and Hsp90, and analyzed by LiCor quantitative imager. (C) HeLa cells were co-transfected with Vpu or indicated Vpu mutant and a GFP expression construct. Cell surface staining for endogenous tetherin was analyzed by flow cytometry 48 h post transfection, as in Figure 2D. (TIF) [file ppat.1002609.s002.tif]

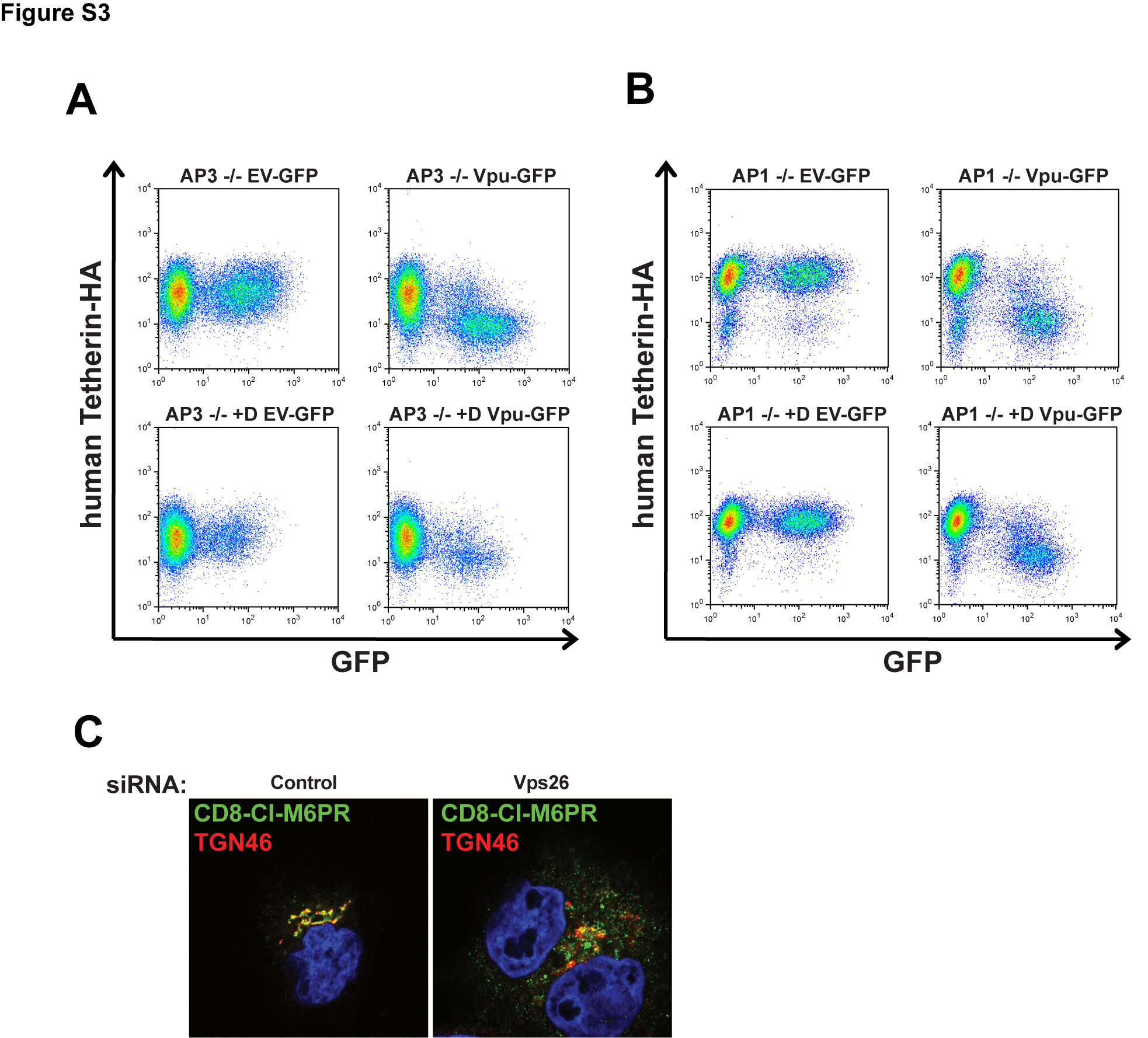

Supplement: Figure S3 — Effects of Vpu on surface expression of human tetherin in murine fibroblasts deficient for AP3 or AP1. Fibroblasts from pearl (AP3δ−/−) (A) or AP-1μ1A−/− mice (B) or their reconstituted counterparts were transduced to express human tetherin bearing an extracellular HA-tag. The cells were then transduced with retroviral vector constructs encoding Vpu linked to GFP via an IRES. 48 h later the cells were surface stained for human tetherin expression using anti-HA antibodies. (C) HeLa-CD8-CI-M6PR cells were treated with control or Vps26 siRNAs. 48 h later the cells were fixed and stained for CD8 (green) and TGN46 (red). (TIF) [file ppat.1002609.s003.tif]

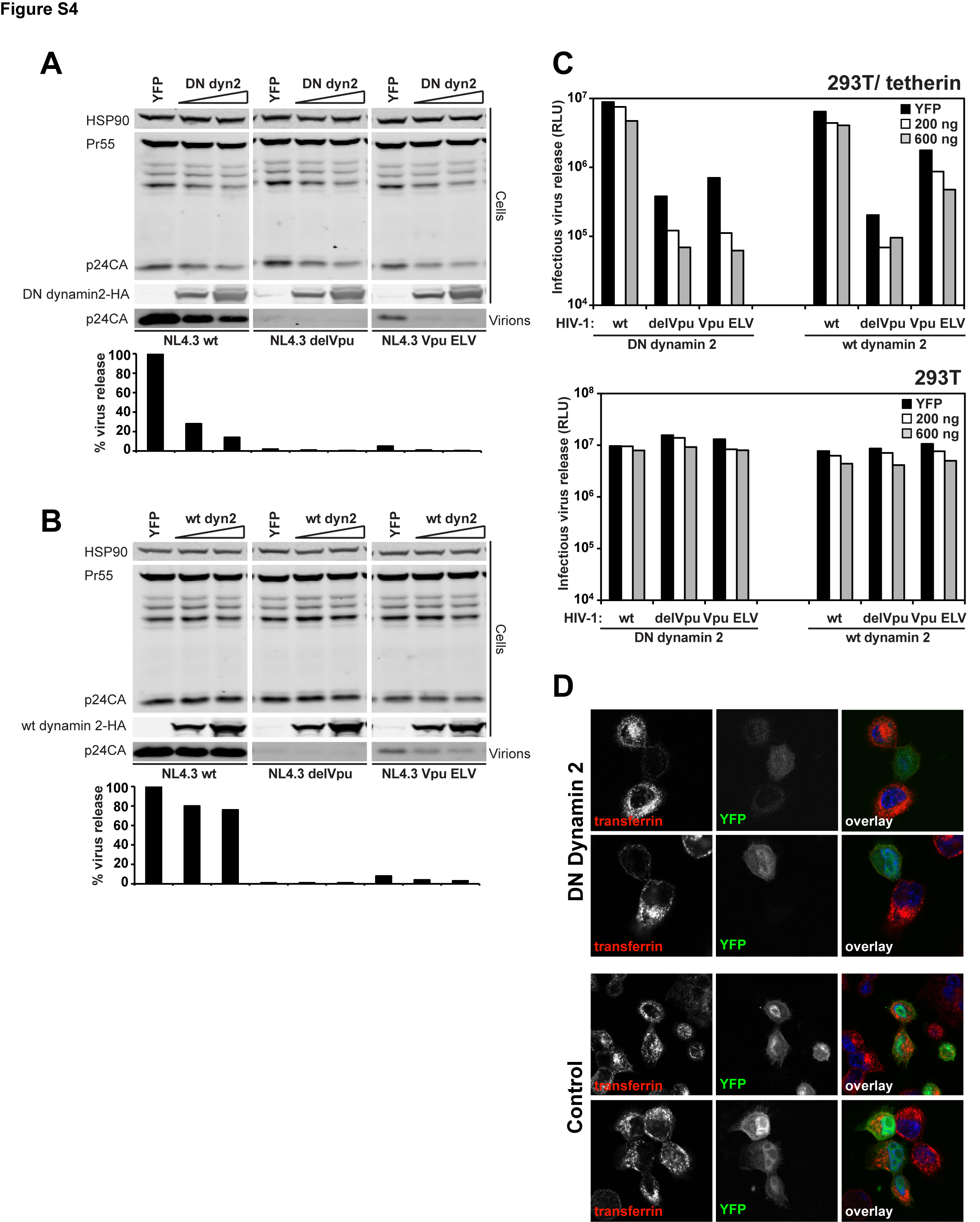

Supplement: Figure S4 — Effects of dominant negative dynamin 2 on Vpu-mediated HIV-1 release. 293T/tetherin cells were transfected with the indicated HIV-1 provirus and increasing doses of HA-tagged dominant negative dynamin 2 (A) or the wildtype protein (B). 48 h later, cell lysates and viral supernatants were harvested and subjected to SDS-PAGE and analyzed by Western blotting for HIV-1 p24CA, dynamin 2-HA and Hsp90, and analyzed by LiCor quantitative imager. Histograms below the blots indicate particle release efficiency compared to wildtype virus release in the absence of dynamin 2 or increasing doses of dynamin 2 expression vector. (C) Corresponding infectivity of viral supernatants from A and B on HeLa-TZM cells alongside those from a parallel experiment in parental 293T cells. (D) 293T/tetherin cells were transfected with pCR3.1 YFP with or without increasing doses of dominant negative dynamin 2. 48 h later the cells were starved in serum-free medium for 30 minutes and then treated for a further 15 minutes with 10 µg/ml Alexa-594-conjugated transferrin, before fixation and imaging. (TIF) [file ppat.1002609.s004.tif]

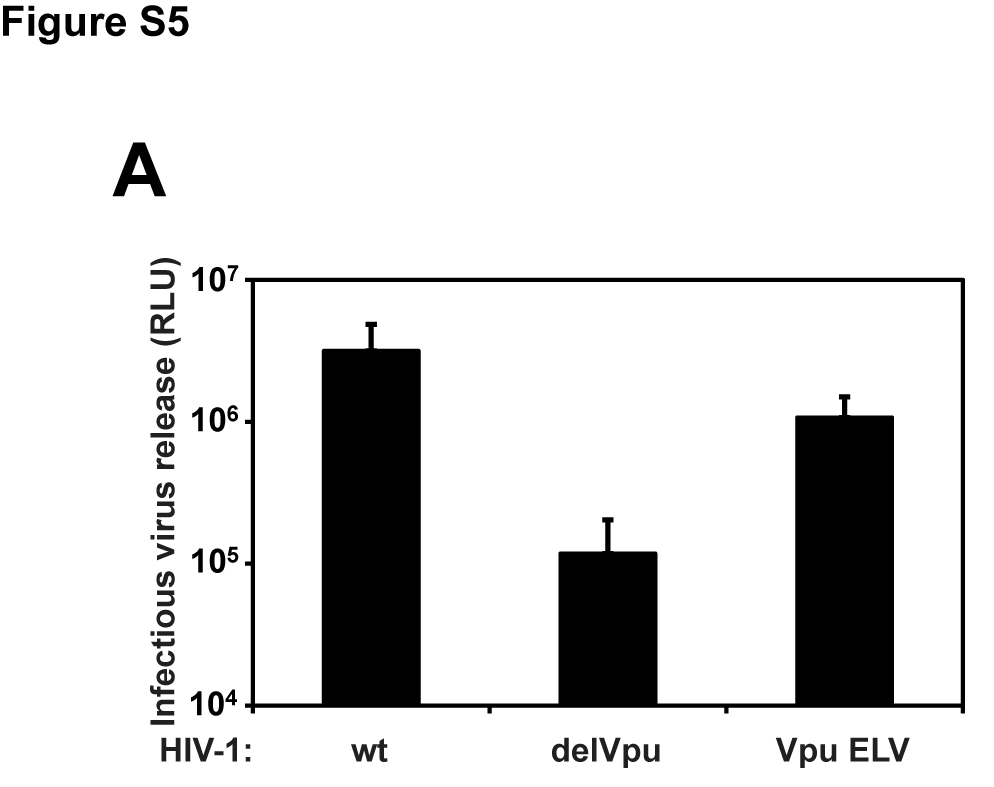

Supplement: Figure S5 — Effect ELV mutant on HIV-1 infectious release from HeLa cells. HeLa cells were infected with VSV-G pseudotyped stocks of the indicated virus at an MOI of 0.5. 48 h later the cell supernatants were harvested and infectivity determined on HeLa-TZM cells. (TIF) [file ppat.1002609.s005.tif]
